# Synthesis, Crystallographic, Quantum Chemical, Antitumor, and Molecular Docking/Dynamic Studies of 4-Hydroxycoumarin-Neurotransmitter Derivatives

**DOI:** 10.3390/ijms23021001

**Published:** 2022-01-17

**Authors:** Dušan S. Dimić, Goran N. Kaluđerović, Edina H. Avdović, Dejan A. Milenković, Marko N. Živanović, Ivan Potočňák, Erika Samoľová, Milena S. Dimitrijević, Luciano Saso, Zoran S. Marković, Jasmina M. Dimitrić Marković

**Affiliations:** 1 Faculty of Physical Chemistry, University of Belgrade, Studentski trg 12-16, 11000 Belgrade, Serbia; markovich@ffh.bg.ac.rs; 2 Department of Engineering and Natural Sciences, University of Applied Sciences Merseburg, Eberhard-Leibnitz-Straße 2, DE-06217 Merseburg, Germany; goran.kaluderovic@hs-merseburg.de; 3 Department of Science, Institute for Information Technologies, University of Kragujevac, Jovana Cvijića bb, 34000 Kragujevac, Serbia; edina.avdovic@pmf.kg.ac.rs (E.H.A.); deki82@kg.ac.rs (D.A.M.); marko.zivanovic@uni.kg.ac.rs (M.N.Ž.); zmarkovic@uni.kg.ac.rs (Z.S.M.); 4 Institute of Chemistry, P. J. Šafárik University in Košice, Moyzesova 11, 04154 Košice, Slovakia; ivan.potocnak@upjs.sk; 5 Institute of Physics of the Czech Academy of Sciences, Na Slovance 2, 182 21 Prague 8, Czech Republic; samolova@fzu.cz; 6 Department of Life Sciences, Institute for Multidisciplinary Research, University of Belgrade, Kneza Višeslava 1, 11030 Belgrade, Serbia; milena.dimitrijevic@imsi.bg.ac.rs; 7 Department of Physiology and Pharmacology “Vittorio Erspamer”, Sapienza University of Rome, P.le Aldo Moro 5, 00185 Rome, Italy; luciano.saso@uniroma1.it

**Keywords:** coumarin, neurotransmitter, molecular docking, molecular dynamics, DFT, X-ray crystallography

## Abstract

In this contribution, four new compounds synthesized from 4-hydroxycoumarin and tyramine/octopamine/norepinephrine/3-methoxytyramine are characterized spectroscopically (IR and NMR), chromatographically (UHPLC-DAD), and structurally at the B3LYP/6-311++G*(d,p) level of theory. The crystal structure of the 4-hydroxycoumarin-octopamine derivative was solved and used as a starting geometry for structural optimization. Along with the previously obtained 4-hydroxycoumarin-dopamine derivative, the intramolecular interactions governing the stability of these compounds were quantified by NBO and QTAIM analyses. Condensed Fukui functions and the HOMO-LUMO gap were calculated and correlated with the number and position of OH groups in the structures. In vitro cytotoxicity experiments were performed to elucidate the possible antitumor activity of the tested substances. For this purpose, four cell lines were selected, namely human colon cancer (HCT-116), human adenocarcinoma (HeLa), human breast cancer (MDA-MB-231), and healthy human lung fibroblast (MRC-5) lines. A significant selectivity towards colorectal carcinoma cells was observed. Molecular docking and molecular dynamics studies with carbonic anhydrase, a prognostic factor in several cancers, complemented the experimental results. The calculated MD binding energies coincided well with the experimental activity, and indicated 4-hydroxycoumarin-dopamine and 4-hydroxycoumarin-3-methoxytyramine as the most active compounds. The ecotoxicology assessment proved that the obtained compounds have a low impact on the daphnia, fish, and green algae population.

## 1. Introduction

Cancer is one of the most prominent causes of death in the modern world. As with most human diseases, it is a very complex, multifactorial, and oxidative stress-related disease. Despite some clinical success of cisplatin complexes, these compounds inflict some deleterious side-effects like myelosuppression, peripheral neuropathy, emesis, nephrotoxicity, fatigue, alopecia, and ototoxicity. They also cause inherent resistance to the treatment of some cancer types [1,2]. Because of this, it is of high importance to create new types of biologically active compounds that are suitable for cancer treatment.

Coumarins are a class of lactones consisting of fused γ-pyrone and benzene rings [3]. They exhibit a significant pharmacokinetic activity as a result of rapid absorption and metabolism in the body. Due to very specific structural features that enable the regulation of a diverse range of cellular pathways, coumarins are considered tumor-selective agents [4]. The modifications of the basic coumarin structure give more complex derivatives that have recently been an inspiration for drug discovery and the drug design process [3]. Their derivatives can easily interact with enzymes and receptors in organisms through weak bond interactions acting as potential medicinal drugs [3]. As anti-cancer agents, they can act on various tumor cells through different mechanisms (inhibition of the telomerase enzyme, downregulation of the oncogene expression, protein kinase activity, and others), expressing both cytostatic and cytotoxic properties [5,6,7,8,9].

Neurotransmitters and their metabolites are naturally occurring compounds in the human body, with pronounced biological activities including neurotransmission, antioxidant activity, and movement, as well as working memory control, attention, sleeping, etc. [10,11]. The blood–brain barrier (BBB) acts as a structural barrier that protects the neurons of the central nervous system and limits the penetration of most newly obtained compounds. Various strategies are applied nowadays to enhance the permeability of active substances [12]. The production of prodrugs, compounds that undergo chemical activation, is one of the simplest ways with a vast potential [13,14]. Coumarin scaffolds are believed to be privileged moieties for the development of the novel classes of biologically active compounds, especially for antineurodegenerative agents [15].

The research presented in this paper implies the design, synthesis, structural characterization, and biological activity evaluation of the new coumarin-neurotransmitter derivatives, namely derivatives of 4-hydroxycoumarin and dopamine, norepinephrine, octopamine, tyramine, and 3-methoxytyramine. The aim of this study is the synthesis and structural characterization of new compounds consisting of 4-hydroxycoumarin and neurotransmitters so as to investigate the possible synergistic effects that these two classes of naturally occurring compounds have on antitumor activity and protein binding. The crystallographic structure of the 4-hydroxycoumarin-octopamine derivative was solved and used as a model for the structural optimization of the other compounds. The theoretical structural characterization includes the optimization of the structure of the obtained compounds based on the crystal structure of the previously described 3-(1-((3,4-dihydroxyphenethyl)amino)ethylidene)-chroman-2,4-dione [16]. The theoretical NMR spectra of the 4-hydroxycoumarin-octopamine derivative were predicted and compared to the experimental ones to verify the applicability of the selected level of theory. Various reactivity parameters, including Fukui functions and HOMO-LUMO gap, are obtained, and the structural moieties responsible for the reactivity are discussed. The cytotoxicity test of the obtained compounds was investigated towards human colon cancer (HCT-116), human adenocarcinoma (HeLa), human breast cancer (MDA-MB-231), and healthy human lung fibroblast (MRC-5) cell lines. The molecular docking and molecular dynamics studies were performed with the carbonic anhydrase IX (hCA-IX). This protein is a member of the α-CA family of enzymes associated with the plasma membrane and participates in the acidification of solid tumors. The significant change of pH is a result of the metabolism shift to anaerobic glycolysis [17]. CAs (EC 4.2.1.1) are zinc-containing metalloenzymes that catalyze the reversible hydration of carbon dioxide in a two-step reaction, to yield bicarbonate and protons [18]. The activity of this protein is a sign of tumor hypoxia and presents a prognostic factor in several human cancers. Selective inhibition of this protein is a potential mode of action for new antiproliferative agents [17,19]. An abnormal increase in CA IX expression in chronic hypoxia and during the development of various cancers contributes to tumorigenesis through at least two mechanisms: pH regulation and cell adhesion control [20]. Previous findings have suggested that coumarin derivatives could potentially act as selective inhibitors of this protein [4,21,22]. The study of Thacker and coworkers showed that 7-hydroxycoumarin derivatives inhibit hCA-IX in the 0.2–9.2 μM range [23]. Additionally, ecotoxicity evaluations for the obtained compounds were performed.

## 2. Results

### 2.1. Chemistry

**Scheme 1:**
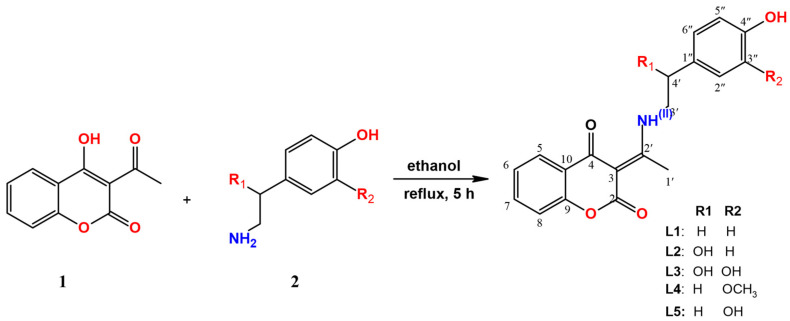
Synthesis of coumarin-neurotransmitters derivatives (**L1**–**L5**) from 3-acetyl-4-hydroxycoumarin (**1**) and respective neurotransmitters and their metabolites (**2**).

The newly synthesized coumarin derivatives were obtained in the reaction of 3-acetyl-4-hydroxycoumarin (1) and neurotransmitters (2) dissolved in methanol as shown in Scheme 1. A similar procedure was applied for the other coumarin derivatives [3,24]. These compounds were characterized by IR and ^1^H and ^13^C NMR spectroscopy. The purity of the compounds was checked by elemental analysis and HPLC. The obtained spectra were mutually very similar, which is a consequence of the structural similarity of the synthesized compounds. Analysis of the IR spectra of compounds **L1**–**L4** shows the bands assigned to the OH and NH groups in the range of 3604–3212 cm^−1^. In the case of **L5**, there was a wide band positioned at 3305 cm^−1^, due to the presence of two OH groups with hydrogen bonds formed and an NH group [16]. The formation of the quasi-six membered ring, O–C–C–C–N–H, with a strong hydrogen bond shifted the band of N–H vibration to lower wavenumbers. This band in similar compounds was joined with the C–H stretching vibration band between 3300 and 3000 cm^−1^ [25,26]. In addition, there were bands assigned to stretching vibrations of lactone C=O and C–O, of close to 1660 and 1200 cm^−1^, respectively. The presence of these vibrations was also shown in the IR spectrum of **L5** at 1668 and 1118 cm^−1^, which proves that the additional OH groups did not induce significant changes in the spectrum [16]. When aminophenol derivatives were attached to the 4-hydroxycoumarin, the stretching carbonyl group vibrations were positioned at 1680 cm^−1^ [27], which is additional proof that the added substituents did not lead to the disruption of the coumarin core planarity [28].

The ^1^H NMR spectra of all of the ligands (**L1**–**L4**; Figures S3, S6, S9 and S12) in DMSO confirm the presence of NH groups, in the form of broad singlets between 13.70–13.83 ppm. Broad signals originating from the hydroxy H atoms were found at slightly lower chemical shifts, between 8.80–9.37 ppm. The NH and OH protons were observed in the ^1^H NMR spectrum of **L5** at 13.66 and 8.81 ppm, respectively. The NH protons in these compounds were positioned at lower chemical shifts than in the 4-hydroxycoumarin aminophenol derivatives (15.2 ppm) [27]. The NMR spectra of these compounds clearly show multiple resonant maxima of aromatic protons of 2,4-dioxochroman and neurotransmitters in the range of 6.69–7.95 ppm. In addition, H atoms at positions C1′, C3′, and C4′ gave resonance at significantly lower frequencies (2.57–3.79 ppm). The hydrogen atom signals of a methyl group (C1′) and C2″H were positioned at almost identical chemical shifts in similar derivatives [25,27]. The ^13^C NMR spectra of neurotransmitter-coumarin derivatives (Figures S4, S7, S10 and S13) indicate the presence of *sp*^2^ hybridized carbon atoms of the coumarin and neurotransmitter moieties in the range of 96.1–161.9 ppm. The chemical shift corresponding to the carbon atom at the C4 position of all of the synthesized compounds was positioned close to 179.0 ppm, while the resonance assigned to C2′ appeared at a slightly lower chemical shift, close to 176.0 ppm. Resonances at significantly lower chemical shifts (18.6–70.3 ppm) indicated *sp*^3^ hybridized carbon atoms (C1′, C3′, and C4′). The carbon atom signals in the ^13^C NMR spectrum of **L5** were almost identical to the presented values [16]. The carbon atom that was part of enclosed ring structures in (E)-3-(1-((2-hydroxyphenyl)amino)ethylidene)-2,4-dioxochroman-7-yl acetate and its isomers were located at 179.60 (C4), 176.02 (C1′), and 168.68 (C3) ppm, which again proves that the electronic density of this part of the molecule was not influenced by other substituents [27].

The HPLC purities were 99.34 (**L1**), 97.70 (**L2**), 96.25 (**L3**), 91.84 (**L4**), and 97.37% (**L5**) (Tables S3–S7 and Figures S5, S8, S11, S14 and S15), which proves that chemically pure compounds were obtained. The retention times for 4-hydroxycoumarin (Figure S1 and Table S1) and 3-acetyl-4-hydroxycoumarin (Figure S2 and Table S2) were 9.36 and 12.33 min, respectively. The retention times for the coumarin-neurotransmitter derivatives were between 10.03 and 12.55 min.

### 2.2. Crystallographic Structure of ***L2***

**Figure 1:**
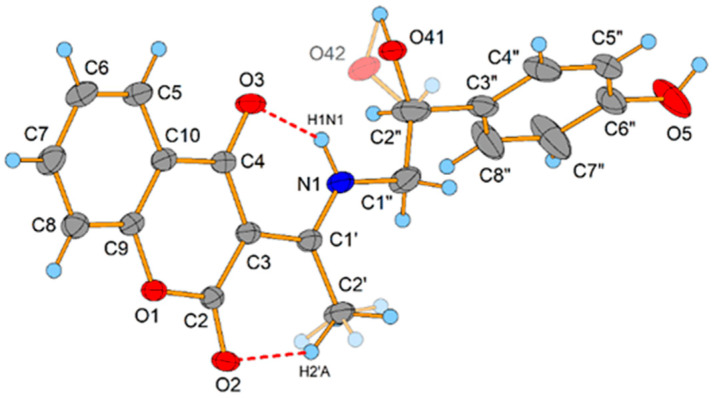
Molecular structure with the atom numbering scheme of **L2**. Displacement ellipsoids are drawn at 50% probability; hydrogen bonds are shown as red dashed lines. The disordered parts of hydroxy and methyl groups are shown in lighter colors.

The X-ray structure analysis revealed that **L2** crystallizes in the triclinic *P*-1 space group (Table 1). Its molecular structure consists of bicyclic coumarin and 2-hydroxy-2-(4-hydroxyphenyl)ethyl fragments joined by an aminoethylidine chain (Figure 1), with hydroxy- (O41) and methyl (C2′) groups disordered over two positions. Within the molecule of **L2**, the dihedral angle between the planes of the coumarin fragment and the 4-hydroxyphenyl ring was 53.05(5)°. The crystallographic structure is presented in Figure 1, while the bond lengths and angles are given in Tables S8 and S9.

A typical structural feature of this type of compound is an intramolecular N−H···O hydrogen bond that forms a six-membered ring with an S(6) graph set motif [29] and thus the molecule occurs in keto-amine tautomeric form. Considering the O3=C4–C3=C1′–N1–H1N1 conjugated bond ring system created owing to the intramolecular hydrogen bond formation, the equalization of the C3–C4 and C3=C1′ (both 1.441(2) Å) bond lengths (Table S8) can be observed, although the bonds are formally single and double, respectively. The same bond lengths were notified in a related 3-(1-((3,4-dihydroxyphenethyl)amino)ethylidene)-chroman-2,4-dione [16]. This can be explained by the π-electron delocalization within the system, and thus it might be concluded that the above hydrogen bond can be classified as a resonance assisted hydrogen bond [30]. Interestingly, in the related 3-(1-((m-toluidine)amino)ethylidene)-chroman-2,4-dione [31] and in 3-(1-(2-hydroxyethylamino)ethylidene)-chroman-2,4-dione [32] compounds, the C3–C4 bond (1.434(2) and 1.430(3) Å, respectively) is even slightly shorter than the C3=C1′ bond (1.436(2) and 1.437(3) Å, respectively). The bond C4=O3 (1.254(2) Å) is elongated when compared to the C2=O2 (1.223(2) Å) bond, which is not involved in a strong hydrogen bond. Very similar bond lengths were shown in related compounds, like 3-(1-((o-toluidine)amino) ethylidene)-chroman-2,4-dione [31], 3-(1-(phenylamino)ethylidene)-chroman-2,4-dione [26], 3-(1-(3-hydroxypropylamino)propylidene)chroman-2,4-dione [24], 2-(1-(2,4-dioxochroman-3-ylidene)ethylamino)-3-methylbutanoate [33], 3-[(1-benzylamino)ethylidene]-2*H*-chromene-2,4(3*H*)-dione [34], or in the 3-[1-((2-hydroxyphenyl)amino)ethylidene)-2*H*-chromene-2,4(3*H*)-dione compound [35]. All other bond lengths and angles (Tables S8 and S9) in the molecule of **L2** were within the normal ranges [36].

**Figure 2:**
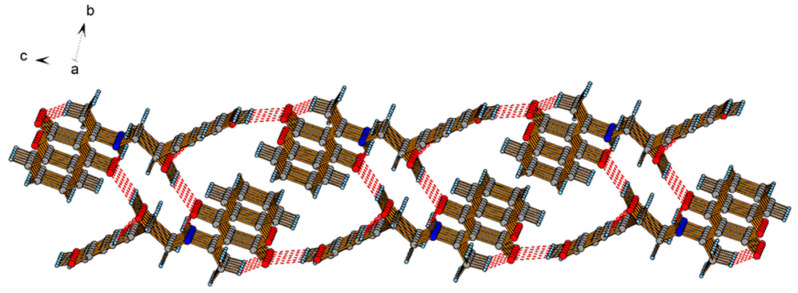
A layer parallel with the *ac* plane in the crystal structure of **L2** is formed by hydrogen bonds (red dashed lines) viewed along the *a*-axis.

Except for the above-discussed N1−H1N1···O3 intramolecular hydrogen bond, due to which the exocyclic C3=C1′ double bond has an *E* geometry, the molecules of **L2** are stabilized in the solid-state by C2′−H2’A···O2 intramolecular and by O−H···O and C−H···O intermolecular hydrogen bonds (Table 2). Due to these bonds, the molecules of **L2** are tied to form layers parallel with the *ac* plane. Within the layers, there are channels along the *a*-axis that are filled by coumarin molecules (Figure 2). These layers are further stabilized by π−π interactions between coplanar phenyl rings of the upper and lower coumarin moieties within the channels. These π−π interactions are characterized by a Cg···Cg^iv^ distance of 3.760(1) Å (iv = −*x*, 1 − *y*, −*z*) and by a γ angle of 26.4° between the normal of the phenyl ring and Cg···Cg vector.

### 2.3. Optimization of an ***L2*** Structure

The obtained crystallographic structure was further used as the starting one for the optimization, as this is the recommended way to obtain a reliable structure for the theoretical studies. Several common functionals (CAM-B3LYP, B3LYP-D3BJ, M05-2X, and M06-2X) in conjunction with the 6-311++G(d,p) level of theory were employed to find a suitable level of theory for the representation of the experimental structure. The bond lengths and angles for the optimized structures at various levels of theory are presented in Tables S8 and S9. Two parameters, namely correlation coefficient (R) and mean absolute error (MAE) were used for the comparison of the crystallographic and optimized structures. The parameter R describes the correlation between the values, while MAE measures the average absolute difference between the experimental and optimized values. These parameters are given in Table S10.

The values of R and MAE prove that all of the optimized structures describe the experimental one well. The correlation coefficient for the bond lengths was 0.97 for all of the functionals, while the MAEs were between 0.011 and 0.013 Å. On the other hand, the highest value of R when bond angles are concerned was obtained for B3LYP-D3BJ functional (0.964) with the lowest value of MAE (0.72°). Structures optimized by other functionals had R values between 0.954 and 0.960 and MAE values between 0.77 and 0.82°. Therefore, the B3LYP/6-311++G(d,p) level of theory was selected for the optimization of the coumarin-neurotransmitter derivatives’ structures. The selected level of theory was the same as the one previously determined for **L5** [16].

### 2.4. Comparison of Experimental and Theoretical NMR Spectra of ***L2***

The applicability of the chosen level of theory can also be proven after the comparison between experimental and theoretical NMR spectra of **L2**. The experimental NMR spectra were recorded in DMSO as a solvent (Figures S6 and S7).

The structure of **L2** was reoptimized in DMSO, but there were no significant changes in bond lengths and angles when compared to the structure in a vacuum. The GIAO method was applied for the prediction of the NMR spectra, and the values of chemical shifts were calculated relative to TMS (optimized at the same level of theory). These values were overestimated and the scaling factors were calculated as 0.938 and 0.994 for the ^13^C and ^1^H NMR spectra, respectively. The experimental and scaled chemical shifts are presented in Table 3. When the ^1^H NMR spectrum is concerned, the correlation coefficient between the experimental and theoretical chemical shifts was 0.978, while MAE was 0.3 ppm. As expected, the lowest value of chemical shift was observed for the methyl group in position C2″, which was 2.5 ppm in the experimental and 2.6 ppm in the theoretical spectrum. Two protons in position C1″ had a chemical shift of 3.4. Due to the symmetry, protons in positions C5″ and C7″, as well as C6 and C8, have the same chemical shifts of 6.7 and 7.3, respectively. The highest values of chemical shifts were obtained for hydrogen atoms in the proximity of electronegative atoms, for example, N1–H, C5–H, and C4″–H. The chemical shifts in the ^13^C NMR spectrum were also well-reproduced (R = 0.998 and MAE = 1.4 ppm), which is a consequence of the rigidity of coumarin and the aromatic parts. The lowest values of chemical shifts were obtained for the aliphatic chain carbon atoms (C2′, C1″, C2″, and C3). The aromatic carbon atoms had signals between 115 and 133 ppm in the experimental (113 and 134 ppm in theoretical) spectrum. The proximity of oxygen atoms in positions C9, C6″, C2, and C4 increased the chemical shifts up to 170 ppm. In addition, the presence of nitrogen atoms in the neighborhood of C1′ led to a chemical shift of 162 ppm in the experimental and 159.9 ppm in the theoretical spectrum. These results prove again that the mentioned level of theory can be used for the successful representation of the experimental structure.

### 2.5. Optimization of Other Structures, NBO, and QTAIM Analyses

**Figure 3:**
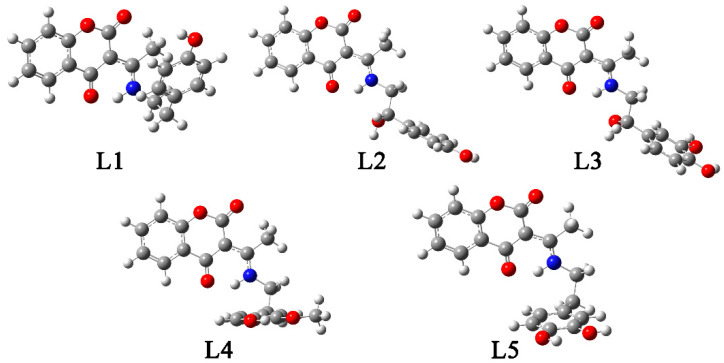
Optimized geometries of the coumarin-neurotransmitter derivatives (at B3LYP-D3BJ/6-311++G(d,p) level of theory).

The structures of other coumarin-neurotransmitter derivatives were optimized at the B3LYP-D3BJ/6-311++G(d,p) level of theory based on the experimental structures of **L2** and **L5**, as previously discussed [16]. These structures consist of a planar coumarin core, aliphatic chain, and aromatic ring with variable substituents. The number of OH groups differs between synthesized compounds, which affects the overall structure. The optimized structures of all of the compounds are presented in Figure 3.

Within each structure, several intramolecular interactions are responsible for its stability, especially within the coumarin core and aromatic rings. These interactions are present in all structures, and are a consequence of the extended delocalization. The interactions in the coumarin core and aromatic ring are not significantly influenced by the additional substituents. Other than that, there is an intermolecular hydrogen bond that encloses a six-membered ring structure (O–C–C–C–N–H), which was previously found in similar structures [31,37], and is discussed within the crystallographic structure. The interaction energies, electron density, and Laplacian for all of the compounds are presented in Table 4. The interaction energy between lone pair on carbonyl oxygen and N–H bond varied between 86 and 102 kJ mol^−1^. These energies were influenced by the presence of the OH group on an aliphatic chain, which lead to a decrease in energy in the case of compounds **L2** and **L3**. When the interaction energies were compared between molecules with catechol and phenol moieties, no significant differences could be observed, which led to the conclusion that these substituents do not disturb the electron distribution in the planar part of the molecule. On the other hand, two OH groups or one OH and methoxy groups led to another stabilization interaction in the form of an intramolecular hydrogen bond with stabilization energy of several kJ mol^−1^. In the case of **L4**, the interaction energy between methoxy and OH groups was equal to 17 kJ mol^−1^. Once two OH groups were present, the interaction energy was 2 (**L3**) and 4 (**L5**) kJ mol^−1^. The electron density and Laplacian values at BCP between the oxygen atom and N–H prove the existence of the interaction that falls between open and closed-shell interactions. The value of the electron density (~0.052 a.u.) and Laplacian (~0.158 a.u.) were lower in the case of **L2** and **L3**, which is in line with the NBO results. Based on the structural considerations, **L2** and **L3** are expected to be less reactive due to the presence of the aliphatic chain OH group.

### 2.6. Reactivity Descriptors

The reactivity descriptors, especially those derived from the energies of HOMO and LUMO orbitals, proved useful for the determination of the reactivity order of structurally similar molecules. In this contribution, the reactivity parameters were calculated for the structures that represent the crystallographic ones well. Molecules with a higher energy of HOMO orbital were more reactive as they were better electron donors. From the data given in Table S11, it is evident that the molecules with the OH group attached to the aliphatic chain had lower energies of HOMO orbitals, which again proves the effectiveness of this group on the overall stability of the coumarin moiety. Based on the HOMO orbital energy, it was expected that **L4** and **L5** would be the most reactive among the obtained compounds. This effect was also reflected in the HOMO-LUMO energy gap, which was higher in the case of compounds **L1**, **L2**, and **L3**, than for the other two compounds 4.5 vs. 4.1 eV (Table S11). This led to the conclusion that the compounds containing dopamine and 3-MT should show a higher activity towards tumor cell lines, which complements previous findings. The dipole moments values are also given in Table S11. These values mostly depend on the overall structure of a molecule, and in the case of **L1**, the value was 3.16 D, which was the lowest among the investigated compounds. For the other compounds, the dipole moment was above 4.8 D. This was expected when the structures were examined. Compounds **L2**–**L4** had an elongated structure, with active groups being positioned on the opposite ends, which significantly contributed to the value of the dipole moment.

The Fukui functions were calculated as given in Supplementary information in order to determine the most probable sites for the electrophilic, nucleophilic, and radical attacks, as these sites are important for the interactions with amino acids, as shown in the proceeding paragraphs. Tables S12–S16 present data on the CFFs for each atom in the structures of the obtained compounds, along with the figures showing the atom numbering schemes (Figures S16–S20). In the following text, only three of the most reactive positions (bold text in Tables S12–S16) are discussed. In compound **L1**, the most active positions for the electrophilic attack are carbonyl oxygen of coumarin moiety (0.077), an oxygen atom attached to the aromatic core (0.57), and a carbon atom that connects the coumarin core and the aliphatic part (0.62). This result was expected as these oxygen atoms are the most electronegative. This also proves the importance of both coumarin core and tyramine moiety for the overall reactivity. For the nucleophilic attack, the carbonyl oxygen atom that is part of the quasi-six-membered ring, and two carbon atoms attached to nitrogen and oxygen, are the most probable sites. The delocalization of electron density within this moiety influences the reactivity of these atoms. Two carbonyl oxygen atoms of the coumarin core are the most reactive towards radicals. In the case of **L2,** the reactive positions were the same, except for the hydrogen atom of the OH group attached to the aromatic core, which was a probable site for the nucleophilic and radical attack. Once the catechol moiety was present in the molecule, as in **L3**, the oxygen atom that acted as a hydrogen-atom donor became a probable site for a nucleophilic attack. The rest of the active positions were the same as in the case of octopamine and tyramine. This shows that the addition of electronegative groups to the structure only partially influenced the reactive positions. In the case of **L4** and **L5**, the same active groups were pointed out by Fukui functions as the most active ones, namely the carbonyl oxygen atoms, carbon atoms attached to the electronegative atoms, oxygen atoms of the catechol moiety, and hydrogen atoms attached to oxygen atoms (Tables S15 and S16). These positions were expected for the strongest interactions with the surrounding amino acids in the following studies.

### 2.7. Cytotoxic Activity

The results from the cytotoxicity investigations are presented in Table 5, as well as the IC_50_ values after 24 and 72 h of treatment and the dose-dependence graphs in Figures S21–S24). In general, the tested compounds showed no cytotoxic effect, with IC_50_ values greater than 500 μM, on metastatic human breast cancer MDA-MB-231 cells and human healthy fibroblasts, MRC-5 cells. On the other hand, the compounds showed a greater or lesser cytotoxic effect on HCT-116 and HeLa cancer cell lines after 72 h of treatment, while the effect after 24 h was neglected. In this sense, all of the tested substances showed a decrease in cell viability on HCT-116 cells in a dose-dependent manner after 72 h. The most significant effect was observed in treatment with **L4** and **L5**, with IC_50_ 74 and 73 µM, respectively. In the case of HeLa cells, **L1** and **L3** showed a low cytotoxicity, while **L2**, **L4**, and **L5** showed a more pronounced effect. Similar to the antitumor effect on HCT-116 cells, **L5** also exerted the most prominent effect (IC_50_ = 92 μM). In addition to analyzing the percentages of viable cells, it was important to examine the relationship between the obtained results, especially those obtained for cancer cells and healthy cells. In this way, it was possible to predict the antitumor potential of certain substances using the Selectivity Index (SI), which considers the selectivity of the tested substances between two model systems [38,39], and it was calculated between HCT-116 and MRC-5 and between HeLa and MRC-5 cells (Table S17). As the compounds do not affect selected cell lines within 24 h of action, the results obtained after 72 h from treatment are discussed. These findings showed a greater selectivity of tested substances towards HCT-116 cells than towards HeLa cells compared to healthy MRC-5 cells. Moreover, greater selectivity in treatment with **L4** and **L5** on HCT-116 cells than on HeLa cells was found. Furthermore, **L3** was selective only towards HCT-116 cells. These results are in good agreement with the herein reported reactivity parameters, which also outline **L4** and **L5** as being the most reactive.

The obtained results show the significant potential of the action on cervical cancer and especially on colorectal cancer cells. Interestingly, the tested compounds did not show a cytotoxic effect on MRC-5 cells, which are extremely sensitive and easy to treat. For this reason, MRC-5 cells were selected as a negative control, as a healthy model system. The effect on MDA-MB-231 cells is not surprising, given their metastatic origin, and thus pronounced resistance to therapy. Based on the presented results, **L4** and **L5** should be subjected to forthcoming research towards colorectal carcinoma cells. To investigate in more detail the possible reasons for the higher reactivity of these two compounds compared to the others, molecular docking and molecular dynamics were performed.

### 2.8. Active Site Confirmation and Molecular Docking Analysis

The protein–ligand interactions between coumarin-neurotransmitter derivatives and amino acids in the active site of the hCA-IX receptor were examined using molecular docking. This protein was taken as a docking model for the explanation of the antitumor experimental activity in several studies [17,19]. Additionally, this protein was chosen because the selected inhibitors (out of seven million compounds) of hCA-IX in the study by Salmas and co-workers showed structural similarities with the obtained compounds [20]. These inhibitors contain one or more aromatic rings, nitrogen atoms, carbonyl, and hydroxyl groups, all of which are present in the structure of coumarin-neurotransmitter compounds [20]. These functional groups are responsible for the strong interactions with the amino acids in the active pocket, namely hydrogen bonds, π−π, and polar. As concluded by Durdagi and co-workers [40], substituted hydroxylic compounds can be used for generating potent hCa-IX inhibitors. The starting neurotransmitters belong to this group of compounds, which was another reason for these theoretical studies. Select coumarin compounds with various substituents inhibit hCa-IX activity in the nanomolar range [4,41]. Therefore, it was of utmost interest to investigate the synergistic effect of these two classes of compounds for the inhibition of this important protein.

**Figure 4:**
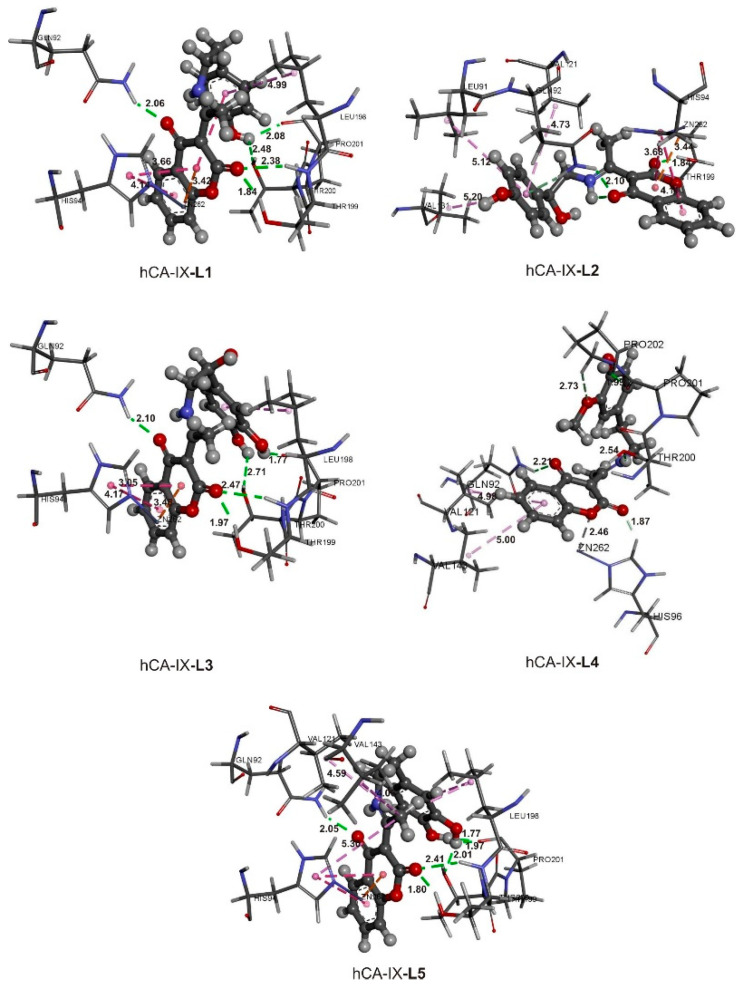
Predicted binding interactions between the investigated molecules and the hCA-IX receptor.

The bound native ligand (5-Acetamido-1,3,4-thiadiazole-2-sulfonamide, **AZA**) was extracted from the hCA-IX receptor, and binding pocket analysis was carried out. After that, the coumarin-neurotransmitter derivatives were docked at the binding site of the hCA-IX receptor to generate the same docking pose as found in its co-crystallized form. This step was done to compare the theoretical binding affinity of the investigated compounds and to correlate it with the experimental inhibition constants for structurally similar polyphenolic compounds [40]. The obtained results of molecular docking studies revealed that investigated compounds were bound to the same amino acids (GLN92, GLU106, LEU198, THR199, THR200, and TRP209) in the active site of hCA-IX as the native ligand. These results suggest that these compounds might also similarly inhibit the activity of hCA-IX as **AZA**. The most stable docking conformations of these compounds are presented in Figure 4, while the most important thermodynamic parameters are listed in Table 6. Several non-covalent interactions exist between the investigated molecules and target receptors. The most prominent interactions are hydrogen bonds, π−cation, π−π, and alkyl−π interactions (Figure 4). The amino acids, such as GLN, THR, and PRO in positions 92, 199, and 200, and 201, respectively, in the primary structure of the hCA-IX receptor chain have a predominant role as the active site of this receptor regarding the investigated ligands. These amino acids form strong hydrogen bonds (bond lengths range from 1.77 to 3.56 Å), while LEU91, HIS94, VAL121, VAL143, and LEU198 form weak π−cation, π−π, and alkyl−π interactions with the Zn ion and aromatic ring of the investigated ligands (Figure 4). It should be noted that the zinc ion in hCA-IX is located in the active site and the neighborhood of the inhibitor. The zinc ion is coordinated by three conserved His residues in a tetrahedral geometry, with H_2_O or OH^−^ as the fourth ligand, which plays a critical role in the hCA-IX inhibitory processes [40]. In the study of Nemr and coworkers, the benzenesulfonamide derivatives showed a significant experimental activity towards hCA-IX, and they were bound to the same group of amino acids (HIS94, THR200, and GLU106). The modified thiadiazole sulfonamide derivatives formed hydrogen bonds with GLN92 and THR200, and hydrophobic interactions with HIS94, VAL121, and LEU199 [19]. These results compare quite well with the presented ones from this study. The interaction formed between the p-chlorophenyl moiety of (3-(5-(4-Chlorophenyl)-1,3,4-thiadiazol-2-yl)ureido)benzenesulfonamide and LEU91 was responsible for the enhanced activity of this compound compared to similar ones, as concluded in the study by [42]. This interaction is observed between coumarin-neurotransmitter derivatives and hCA-IX, as previously discussed.

The results given in Table 6 present almost the same trends in the binding affinities of the investigated molecules towards hCA-IX, with the actual binding free energies values being different for 2–4 kJ mol^−1^. The highest binding energies were obtained for the derivatives with 3-methoxytyramine (−42.1 kJ mol^−1^) and dopamine (−43.7 kJ mol^−1^). Again, these two derivatives were proven to be the most reactive ones, and this result complements the findings from previous sections. These values were higher by several kJ mol^−1^ than for **AZA**, which reproduces the previous docking studies including this native ligand well [17]. This result was expected, as **AZA** also contains several active positions bearing electronegative nitrogen and sulfur atoms. The computed values of the inhibition constants between these compounds and the hCA-IX receptor were in the same range as the experimentally observed inhibition constants in the literature [40], which proves that a comparable system was obtained in this study. The inhibitory effect of these compounds was similar to that of the naturally occurring polyphenols, for example, resorcinol [40].

From the results in Table 6, it can be seen that the largest quantitative contribution to ΔG_bind_ was derived from ΔG_vdw+hbond+desolv_, which includes the conventional hydrogen bonds and van der Waals interactions. Careful examination of the interactions shows that the positions that were determined by the Fukui functions as the most reactive ones were indeed included in these interactions. Compounds **L3**, **L4**, and **L5** had the highest absolute values of this parameter due to the presence of two oxygen atoms directly attached to the aromatic rings. The electrostatic interactions were more significant for hCA-IX-**L5** than for other protein–ligand complexes and they were reflected in the relatively high contribution of ΔG_elec_ in ΔG_bind_. The torsional energy was between 4.8 and 6.7 kJ mol^−1^, which shows that rotation within the structure between the coumarin core and aromatic ring could influence the binding energy to a certain extend.

### 2.9. Molecular Dynamics

**Figure 5:**
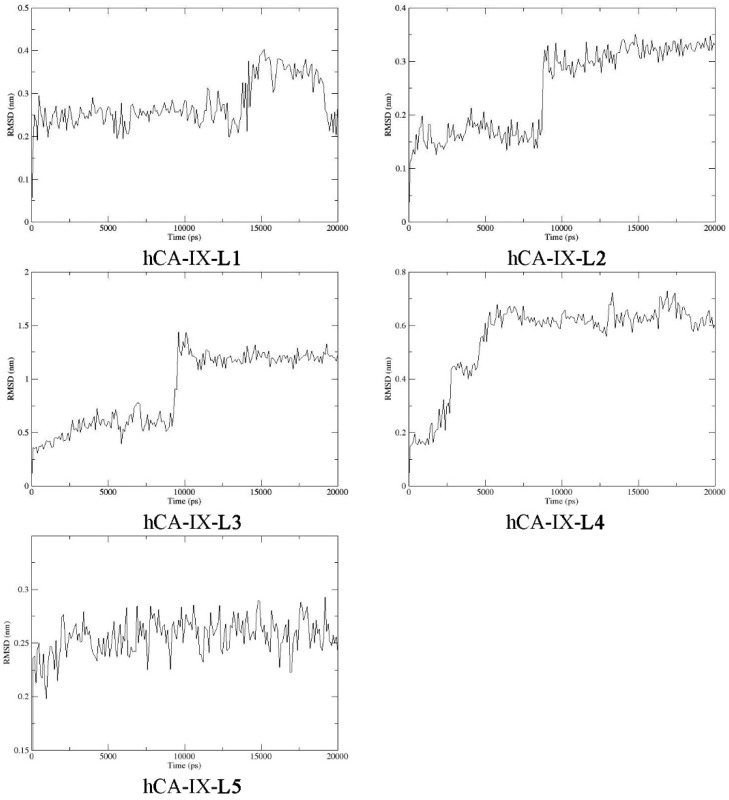
The plot of root mean square deviation (RMSD) of the C–Cα–N backbone vs. simulation time for solvated hCA-IX in complex with all coumarin-neurotransmitter derivatives during 20 ns molecular dynamics simulations.

The molecular dynamic analyses were performed with tools in GROMACS (Root Mean Square Deviation (RMSD), Root Mean Square Fluctuation (RMSF), and Radius of gyration (Rg)) to examine the system properties, including the overall stability, local residue, and general structure fluctuations through the simulations. The direct changes in the protein conformation from the initial coordinates could be measured by the RMSD (Figure 5). These values of the protein backbone in complex with all of the investigated molecules were computed for the initial structure as a frame reference (0 to 20 ns). The RMSD value for **L5** steadily increased from 0 to 3 ns and reached an equilibration that remained throughout the simulation period. On the other hand, the RMSD values for other ligands showed oscillations during the simulation period, indicating that the compound was adapting another confirmation within the binding pocket (Figure 5). The longest times (>10,000 ps) required to reach the equilibration were obtained for **L1**, **L2**, and **L3**, which reflects the stability of the starting structures. The average RMSD values for the ligands were 0.27 ± 0.06, 0.24 ± 0.08, 0.89 ± 0.34, 0.55 ± 0.15, and 0.25 ± 0.02 Å (**L1**, **L2**, **L3**, **L4**, and **L5**, respectively).

**Figure 6:**
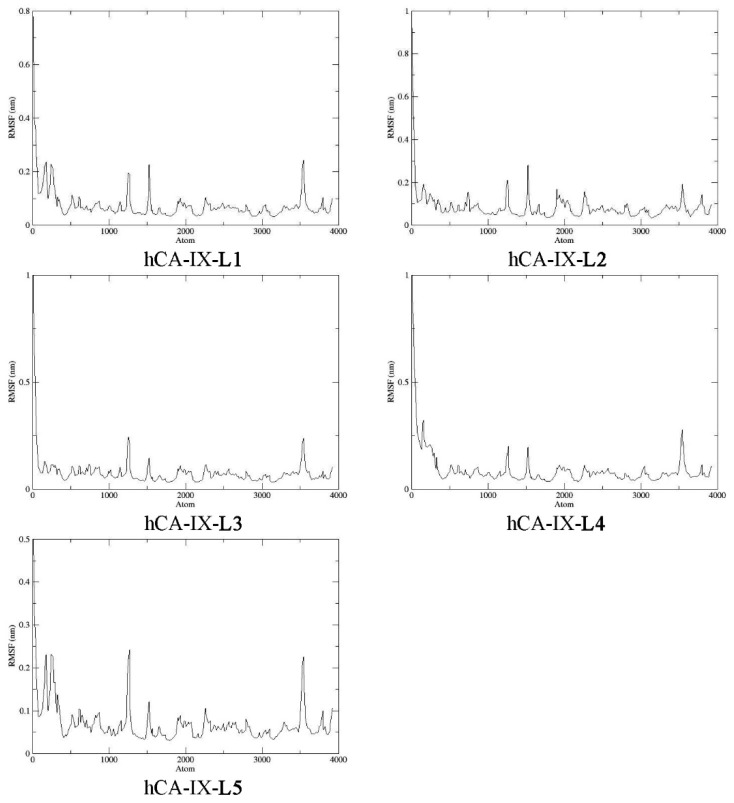
The root mean square fluctuation (RMSF) values of hCA-IX in complex with the investigated candidate compounds plotted against residue numbers.

To explore the local protein flexibility, the time average of RMSF values of the 3920 amino acids of the hCA-IX protein in the presence of all ligands over the simulation period was calculated (Figure 6). Flexibility is an important property in protein function, and more flexible proteins would have enlarged binding pockets, which significantly influences the substrate-product kinetics and affinity [43] (Figure 6). The average RMSF values were almost the same, at 0.09 ± 0.01 nm for all of the investigated compounds. The flexibility of the protein was reduced in all of the investigated complexes, but the values were not significantly different due to the interactions with the active positions. This points out the weak interactions as the main binding mode of the obtained coumarin-neurotransmitter derivatives.

The radius of gyration (Rg) of the protein is associated with its size and compactness. The Rg values of all of the complexes were found to be 1.75 nm at the initial state (Figure S25). The Rg values of the complex between a protein with **L5** and **L3** were stabilized after the initial increase at 2.5 ns, supporting that the systems reached an equilibrium state. The change in Rg value for protein–ligand complex with **L4** was much more complex, with an increase until 6 ns and then a slight decrease until 15 ns. On the other hand, the Rg value for **L2** decreased from 5 ns to 12 ns, and then the system reached an equilibrium state. The MD simulation results confirmed the stability of all of the investigated compounds at the active site of the target receptor.

The obtained docking complexes were further subjected to MD energy contribution analysis via MM/PBSA protocol [44], based on van der Waals, electrostatic, polar solvation, and nonpolar solvation energies. The values of various contributions to the total energy are presented in Table 7.

As listed in Table 7, the binding free energies of **L5** and **L4** were significantly higher in absolute value than those for the other ligands, at −189.7 and −184.9 kJ mol^−1^, respectively. This result indicates a higher binding affinity towards hCA-IX than for **L1** (−68.4 kJ mol-1), **L2** (−47.8 kJ mol^−1^), and **L3** (−75.9 kJ mol^−1^). A detailed decomposition of the energy components reveals that the contribution of the energy of electrostatic interactions (ΔE_elec_) to the total binding free energy for hCA-IX-**L5**, hCA-IX-**L1**, and hCA-IX-**L2**, when compared to the other two complexes, was much more prominent. The value of ΔG_polar_ negatively contributed to the binding process and it was much higher in these three complexes than in the hCA-IX-**L4** and hCA-IX-**L3** complexes. The values of nonpolar free energy for all complexes slightly contributed to the total binding free energy. Based on the obtained results, it can be concluded that the vacuum potential energy (van der Waals and electrostatic interactions) is a major contributor to the total binding free energy. It is also important to point out that the order of binding energies coincides well with the activity towards HCT-116 and HeLa cell lines, thus proving that MD calculations can be beneficial for understanding the underlying mechanism of the cytotoxic activity of compounds.

The contribution of the specific residues to the overall binding energy is given in Figure S26. As seen in this figure, the positive contributions were recorded for the hCA-IX-**L4** and hCA-IX-**L5** complexes. The main contributions came from HIS94, GLU106, ASP132, and THR199, some of which were pointed out in the previous section as the amino acids in the active pocket of hCA-IX responsible for binding to **AZA**.

This result again proves the applicability of the chosen model system for the cytotoxicity analysis. The negative contributions were calculated for TRP5, HIS64, GLN92, THR200, and PRO201 in the hCA-IX-**L4** complex and LEU198 and PRO202 in the hCA-IX-**L5** complex. Bearing in mind that only these complexes had a positive and negative contribution to free binding energy, this is why **L5** and **L4** showed a higher affinity to the hCA-IX receptor than the other ligands (Table 7). These results are in agreement with the results obtained by molecular docking, as well as with the experimental results. The significant negative contributions are recorded for interactions with GLU106 in hCA-IX-**L1** and hCA-IX-**L2** complexes, and these interactions may be the reason for the lower affinity towards hCA-IX (Table 7).

### 2.10. Ecotoxicology Assessment

In the framework of the potential antitumor activity, the Lipinski rules were applied to the newly obtained derivatives. All coumarin-neurotransmitter derivatives passed the Lipinski rules (Table S18). The ecotoxicology assessment of the obtained compounds was predicted towards fish, daphnia, and green algae. Special emphasis was put on the investigation of the substituent effect on these parameters. The values for the acute and chronic toxicity are presented in Table 8.

The obtained results for the ecotoxicity evaluation showed different effects of the obtained coumarin derivatives on the selected organisms. When acute toxicity on fish and daphnia were concerned, all of the investigated substances fell within the category of not harmful substances with LC50 > 100 mg L^−1^. In the case of green algae, **L1**, **L4**, and **L5** belonged to harmful substances. **L2** (EC_50_ = 399 mg L^−1^) and **L3** (EC_50_ = 109 mg L^−1^) were still in a not harmful category. The reason is probably in the aliphatic OH group that additionally disrupts the delocalization within a structure. The chronic toxicity of the obtained compounds was within th limits for the not harmful category, with ChV > 10 mg L^−1^, except in the case of **L1** (ChV, daphnia = 8.2 mg L^−1^) and **L5** (ChV, green algae = 3.18 mg L^−1^). Again, the actual values of ChV were much higher in the case of **L2** and **L3** than for the other three molecules. The results of this study prove the low ecotoxicity impact of the obtained compounds and open the possibility for further experimental studies.

## 3. Materials and Methods

### 3.1. Chemical Reagents and Instruments

Chemicals (purity > 98%) used for the synthesis were acquired from Merck. Compound 3-acetyl-4-hydroxycoumarin (1) was prepared according to a previously reported method [45].

IR spectroscopy measurements were performed on a Perkin-Elmer Spectrum One FT-IR spectrometer using the KBr pellet technique. The NMR spectra were recorded on a Varian Gemini spectrometer (200 MHz for ^1^H and 50 MHz for ^13^C) in DMSO-d6. Elemental microanalysis for carbon, hydrogen, and nitrogen was done in the Centre for Instrumental Analysis, at the Faculty of Chemistry, University of Belgrade. Elemental (C, H, and N) analysis of the samples was carried out on an Elemental analysis system VARIO EL III CHNOS, model—Elementar Analysensysteme GmbH, 2003.

### 3.2. Synthesis of Coumarin- Neurotransmitters Derivatives

The newly synthesized coumarin derivatives were obtained by reacting 3-acetyl-4-hydroxycoumarin (1) (1 mmol) and neurotransmitters (2) (2 mmol), dissolved in methanol (50 mL), and stirred at reflux for 5 h. The progress of the reaction was monitored by thin-layer chromatography (TLC). When the reaction was complete, the resulting mixture was cooled to room temperature and the precipitate was collected by filtration. The obtained products were later purified from ethanol. All newly synthesized compounds were characterized by elemental analysis, ^1^H NMR, ^13C^ NMR, and IR spectroscopy. The synthesis and structural characterization of another compound included in the study, namely 3-(1-((3,4-dihydroxyphenethyl)amino)ethylidene)-chroman-2,4-dione, was previously described [16]. Throughout the manuscript, this compound is denoted as **L5**.

#### 3.2.1. (E)-3-(1-((4-hydroxyphenethyl)amino)ethylidene)chromane-2,4-dione (**L1**) 

White powder. Yield: 0.52 g (81%). HPLC purity: 99.34%. *Anal. Calc.* For C_19_H_17_O_4_N (Mr = 323.12) %: C, 70.58; H, 5.30; N, 4.33. Found: C, 70.75; H, 5.24; N, 4.76. IR (KBr) ν cm^−1^: 3223 (OH, NH), 3019 (ArC-H), 2903 and 2848 (CH), 1661 (C=O), 1610, 1573, 1513 (C=C), 1466 (ArC-C), 1229 (C-O). ^1^H NMR (200 MHz, DMSO-d_6_) δ ppm: 2.57 (s, 3H, C–1′), 2.87 (t, J = 7.1Hz, 2H, C–4′), 3.79 (q, J = 6.8 Hz, 2H, C–3′), 6.71 (m, 2H, Ar-H), 7.11 (m, 2H, Ar-H), 7.26 (m, 2H, C–6, C–8), 7.62 (m, J = 8.2, 7.3Hz, 1H, C–7), 7.92 (m, J = 7.8, 1.7 Hz,1H, C–5), 9.29 (s, 1H, OH), 13.69 (s, 1H, NH). ^13^C NMR (50 MHz, DMSO-d_6_) δ ppm: 18.3 (C–1′), 33.8 (C–4′), 45.6(C–3′), 96.0 (C–3), 115.4 (C–3″, C–5″), 116.2 (C–8), 120.3 (C–10), 123.64 (C–6), 125.7 (C–5), 127.9 (C–2″, C–6″), 129.8 (C–1″), 133.9 (C–7), 153.0 (C–9), 156.0 (C–4″), 161.9 (C–2), 176.1 (C–2′), 179.48 (C–4).

#### 3.2.2. (E)-3-(1-((2-hydroxy-2-(4-hydroxyphenyl)ethyl)amino)ethylidene)chromane-2,4-dione (**L2**)

Yellow powder. Yield: 0.50 g (73 %). HPLC purity: 97.70%. *Anal. Calc*. For C_19_H_17_O_5_N (Mr = 339.11) %: C, 67.25; H, 5.05; N, 4.13. Found: C, 67.55; H, 5.24; N, 4.46. IR (KBr) ν cm^−1^: 3444 and 3267 (OH, NH), 3047, 3019 (ArC-H), 2922 and 2873 (CH), 1676 (C=O), 1606, 1516 (C=C), 1462 (ArC-C), 1220 (C-O). ^1^H NMR (200 MHz, DMSO-d_6_) δ ppm: 2.59 (s, 3H, C–1′), 3.71 (dtd, J = 21.7, 13.8, 5.1 Hz, 2H, C–3′), 4.81 (dd, J = 7.8, 3.8 Hz, 1H), 5.82 (s, 1H, OH), 6.92–6.58 (m, 2H, Ar-H), 7.08–7.37 (m, 4H, Ar-H), 7.62 (ddd, J = 8.3, 7.3, 1.7 Hz, 1H, C–6), 7.95 (dd, J = 7.8, 1.7 Hz, 1H, C–5), 9.37 (s, 1H, OH), 13.83 (s, 1H, NH). ^13^C NMR (50 MHz, DMSO-d_6_) δ ppm: 18.6 (C–1′), 51.6 (C–3′), 70.2 (C–4′), 96.2 (C–3), 115.0 (C–3″, C–5″), 116.2 (C–8), 120.4 (C–10), 123.6 (C–6), 125.7 (C–5), 127.3 (C–2″, C–6″), 132.7 (C–1″), 133.9 (C–7), 153.1 (C–9), 156.8 (C–4″), 162.0 (C–2), 176.0 (C–2′), 179.42 (C–4).

#### 3.2.3. (E)-3-(1-((2-(3,4-dihydroxyphenyl)-2-hydroxyethyl)amino)ethylidene)chromane-2,4-dione (**L3**)

Beige powder. Yield: 0.55 g (77%). HPLC purity: 96.25%. *Anal. Calc*. For C_19_H_17_O_6_N (Mr = 355.11) %: C, 64.22; H, 4.82; N, 3.94. Found: C, 64.45; H, 4.54; N, 4.16. IR (KBr) ν cm^−1^: 3604, 3212 (OH, NH), 1666 (C=O), 1610, 1559, 1530 (C=C), 1468 (ArC-C), 1232 (C-O). ^1^H NMR (200 MHz, DMSO-d_6_) δ ppm: 2.59 (s, 3H, C–1′), 3.87–3.49 (m, 2H, C–4′), 4.79–4.66 (m, 1H, C–4′), 5.80 (d, J = 4.1 Hz, 1H, OH), 6.69 (d, J = 1.5 Hz, 2H, Ar-H), 6.84 (d, J = 1.4 Hz, 1H, Ar-H), 7.18–7.95 (m, 4H, Ar-H), 8.88 (d, J = 7.0 Hz, 2H, OH), 13.81 (s, 1H, NH). ^13^C NMR (50 MHz, DMSO-d_6_) δ ppm: 18.6 (C–1′), 51.7 (C–3′), 70.3 (C–4′), 96.2 (C–3), 113.7 (C–2″), 115.3 (C–5″), 116.3 (C–8), 117.0 (C–6″), 120.4 (C–6), 123.7 (C–5), 125.8 (C–7), 133.4 (C–1″), 133.9 (C–7), 145.1 (C–4″), 144.7 (C–3″), 153.1 (C–9), 162.1 (C–2), 175.9 (C–2′), 179.4 (C–4).

#### 3.2.4. (E)-3-(1-((4-hydroxy-3-methoxyphenethyl)amino)ethylidene)chromane-2,4-dione (**L4**)

White powder. Yield: 0.57g (81%). HPLC purity: 91.84%. *Anal. Calc*. For C_20_H_19_O_5_N (Mr = 353.13) %: C, 67.98; H, 5.42; N, 3.96. Found: C, 68.34; H, 5.14; N, 4.26. IR (KBr) ν cm^−1^: 3559, 3267 (OH, NH), 3055 (ArC-H), 2940, 2917 (CH), 1666 (C=O), 1613, 1591 (C=C), 1468 (ArC-C), 1279 (C-O). ^1^H NMR(200 MHz, DMSO-d_6_) δ ppm: 2.57 (s, 3H, C–1′), 2.88 (t, J = 6.9 Hz, 2H, C–4′), 3.76 (s, 3H, OCH_3_), 3.83 (t, J = 6.4 Hz, 2H, C–3′), 6.70 (d, J = 1.0 Hz, 2H, C–6, C–7), 6.91 (s, 1H, C–8), 7.17–7.92 (m, 4H, Ar-H), 8.82 (s, 1H, OH), 13.70 (s, 1H, NH). ^13^C NMR (50 MHz, DMSO-d_6_) δ ppm: 18.4 (C–1′), 34.2 (C–4′), 45.8 (C–3′), 45.8 (OCH_3_), 96.1 (C–3), 113.3 (C–2″), 115.6 (C–5″), 116.3 (C–8), 120.3 (C–10), 121.2 (C–6″), 123.7 (C–5), 125.7 (C–5), 128.6 (C–1″), 134.0 (C–7), 145.3 (C–4″), 147.6 (C–3″), 153.1 (C–9), 156.0 (C–4″), 161.9 (C–2′), 176.2 (C–2), 179.5 (C–4).

### 3.3. UHPLC-DAD Analysis

Determination and quantification of the components were performed using a Dionex Ultimate 3000 UHPLC system equipped with a diode array detector (DAD) (ThermoFisher Scientific, Basel, Switzerland). The elution was performed at 40 °C on a LunaC8(2) column (100 × 4.6 mm) with a 3.0 μm particle size (Phenomenex, Torrance, CA, USA). The mobile phase consisted of (A) 0.1% aqueous formic acid solution and (B) 0.1% acetonitrile formic acid solution, which was applied in the following gradient elution: 5% B in the first min, 5–95% B for 1.0–14.0 min, from 95% to 5% B at 14.0 min and 5% B until 20.4 min. The flow rate was set to 0.5 mL min^−1^ and the detection wavelengths to 254 and 280 nm. The injection volume was 10 μL. Samples were filtered through a 0.45 mL membrane filter before being analyzed.

Xcalibur software 2.2 (ThermoFisher Scientific, Bremen, Germany) was used for the instrument control. The compounds were quantitated by a set peak detection algorithm and direct plot *ICIS*.

A stock solution of each ingredient was prepared in DMSO (4-hydroxycoumarin 0.75 mg/1 mL; 3-acetyl-hydroxycoumarin 0.65 mg/1 mL; **L1** 1.8 mg/1 mL; **L2** 1.86/1 mL; **L3** 2 mg/1 mL; **L4** 1.75 mg/1 mL; and **L5** 1.86/1 mL;). Dilution of the stock solution with methanol was always done 10 times.

### 3.4. X-ray Data Collection and Structure Refinement

The data collection for **L2** was carried out on a SuperNova diffractometer from Rigaku OD equipped with an Atlas2 CCD detector. Crysalis CCD was used for data collection while Crysalis RED [46] was used for cell refinement, data reduction, and absorption correction. The structure was solved by SHELXT [47] and subsequent Fourier syntheses using SHELXL-2018 [48], implemented in the WinGX program suit [49]. The analysis of bond distances and angles, as well as the hydrogen bonds, was performed using SHELXL-2018, while PLATON [50] running under WinGX was used to analyze the π–π interactions. DIAMOND [51] was used for the molecular graphics. A summary of the crystal data and structure refinement for **L2** is presented in Table 1.

### 3.5. Cell Culturing

The in vitro cytotoxic activity of the tested compounds was investigated on four model systems: human colorectal carcinoma HCT-116, human breast cancer MDA-MB-231, human cervical cancer HeLa, and human healthy fibroblast MRC-5 cell lines. The cell lines of low passages were purchased from ECACC and were cultivated in Dulbecco’s Modified Eagle Medium (DMEM; Sigma-Aldrich, Germany, D5796) supplemented with 10% fetal bovine serum (Sigma-Aldrich, F4135-500ML) and 1% penicillin/streptomycin (Sigma-Aldrich, P4333-100ML) in 75 cm^2^ culture flasks. The cells were harvested in an incubator under a humidified atmosphere with 5% CO_2_ at a physiological temperature of 37 °C, and after a few passages and a confluence of about 90%, the cells were used for the MTT assay.

### 3.6. Cytotoxicity Assay

The capacity of tested compounds to prevent the growth of four different cell lines was assessed by a standardized MTT assay (Laboratory for Bioengineering protocol CB-005, Kragujevac, Serbia), based on spectrophotometric measurement of the reduction of 3-(4,5-dimethylthiazol-2-yl)-2,5-diphenyltetrazolium bromide (MTT, Acros Organics, NJ, USA 158990010) to purple formazan crystals subsequently dissolved in dimethyl sulfoxide (DMSO; Fisher Chemical, IL, USA D/4121/PB15). A number of 1 × 10^4^ cells per well (in 96-well microplates) were seeded and maintained in an incubator for 24 h to allow for cell attachment. After the incubation period, the cells were treated with the investigated compounds in the concentration range from 0.1 to 500 µM dissolved in DMEM. Cytotoxic effect was calculated 24 and 72 h from treatment by determination of the number of viable cells. The absorbance measured at 550 nm and was performed on a microplate reader (Rayto 2100C, China). The cytotoxicity was derived as the ratio of the absorbance of the treated cells divided with the absorbance of negative control (untreated cells) and multiplied by 100 to obtain the percentage of the viable cells [38].

### 3.7. Computational Methods

The optimization of the coumarin-neurotransmitter structures was performed in the Gaussian Program Package [52] by the employment of several common functionals (APFD, B3LYP-D3BJ, M05-2X, and M06-2X) in conjunction with the 6-311++G(d,p) basis set [53,54,55,56,57]. The optimization was performed without any geometrical constraints and the number of imaginary frequencies was zero. To mimic the experimental conditions, the Conductor-like Polarizable Continuum (CPCM) solvent model was applied [58]. The NMR spectra of **L2** were predicted by the Gauge Independent Atomic Orbital (GIAO) approach [59,60]. The interactions governing the structures of synthesized derivatives were analyzed in the Natural Bond Orbital (NBO) analysis framework [61]. The stability of structures was also investigated by the Quantum Theory of Atoms in Molecules (QTAIM) approach [62] in the AIMAll program package [63]. This approach is based on the analysis of the electron density and its Laplacian at the Bond Critical Points (BCP). Within this framework, the interactions can be divided into two groups. The first group consists of closed-shell interactions (covalent bonds) with an electron density of 0.1 eA and large negative Laplacian. The second group includes open-shell interactions (ionic bonds, hydrogen bonds, and van der Waals interactions) with an electron density between 0.001 and 0.04 eA and a small positive Laplacian.

### 3.8. Fukui Functions

Fukui functions are very useful parameters when it comes to the investigation of the possible electrophilic, nucleophilic, and radical attack sites. These parameters can also be used for the prediction of the binding sites for the interactions with various amino acids in proteins. Fukui functions describe the change in total electron density when anions and cations are formed. Their evaluation depends on the chosen methodology, and several methods for their calculation are found in the literature [64]. Condensed Fukui functions (CFF) can be determined for the specific atoms in the following way:

(1) $$f_{A}^{+}=q_{N}^{A}-q_{N+1}^{A}$$

(2) $$f_{A}^{-}=q_{N-1}^{A}-q_{N}^{A}$$

(3) $$f_{A}^{0}=\left[q_{N-1}^{A}-q_{N+1}^{A}\right]/2$$

To obtain CFFs, the charges on atom *A* have to be determined in the neutral, anionic, and cationic species. When CFF values are compared, a higher value represents a more reactive site [65]. The CFFs were calculated in the Multiwfn program package [66].

### 3.9. Molecular Docking

The inhibitory effect of the coumarin-neurotransmitter derivatives against the hCA-IX receptor was examined using molecular docking simulations. The binding pockets and sites of the target receptor were determined in the AutoGridFR (AGFR) program [67]. The crystal structure of this receptor was downloaded from RCSB Protein Data Bank in PDB format (PDB ID: 3IAI) [18]. The protein was arranged in Discovery Studio 4.0 [68] for molecular docking. The AutoDockTools (ADT) graphical interface was applied to add the Kollman partial charges and polar hydrogens. The optimization of ligand structures was done by density functional theory at the previously mentioned level and was used further for docking. The structures of the ligands were set to be routable in the ADT program, while the protein was kept as a rigid structure. The Lamarckian Genetic Algorithm (LGA) method was employed for protein–ligand flexible docking. The grid center with dimensions 68.864 × 52.242 × 14.376 Å^3^ in *−x*, *−y*, and *−z*-directions of the hCA-IX receptor was set to cover the protein binding site and to accommodate the ligand to move freely. A grid point spacing of 0.375 Å was employed for the auto grid runs. The AutoDock 4.2 software [69] was used to examine the binding affinity of the investigated compounds. The interactions between the target protein and the investigated compounds were analyzed and illustrated in Discovery Studio 4.0 and AutoDockTools.

### 3.10. Molecular Dynamics

As the starting models for the MD simulations, the hCA-IX-**L1**, hCA-IX-**L2**, hCA-IX-**L3**, hCA-IX-**L4**, and hCA-IX-**L5** docked complexes were used. The parameterization of the title ligands was performed by the CHARMM36m force [70]. The CHARMM-GUI web server [71] was used for the preparation of the protein–ligand structure inputs for equilibration and production. The 0TIP3P solvation model was employed for the solvation of the investigated systems, while the sodium chloride ions were added to neutralize the systems to a salt concentration of 0.15 M in KCl. The neutralized systems were energetically minimized by the steepest descent and conjugated gradient algorithms with up to a tolerance of 1000 kJ mol^−1^ nm^−1^ during 5000 steps. After energy minimization, each system was equilibrated at a human body temperature of 310.15 K using the Berendsen weak coupling method in NVT (constant volume) ensemble condition with a 2 ns time scale. The production MD phase was performed in the NPT ensemble using the LINCS algorithm for a 20 ns time scale, including a modified Berendsen thermostat (τT = 1 ps) and a Parrinello−Rahman barostat (τP = 2 ps) [71,72]. The simulation trajectories were propagated to 20 ns using the GROMACS 5.1.5 package [73]. The *g_mmpbsa* program in conjunction with the GROMACS program coupled with Adaptive Poisson−Boltzmann Solver (APBS) [73,74] was applied to calculate the free energies of binding of the protein–ligand complexes.

### 3.11. Ecotoxicology Assessment

Due to the increased concerns on the ecotoxicology effects of newly introduced drugs, ecotoxicology assessment is an important step in their evaluation. The results are often represented as the acute (LC_50_ and EC_50_) and chronic (CHV) toxicities towards three classes of aquatic organisms, namely fish, daphnia, and green algae. For the prediction of these values, the ECOlogical Structure−Activity Relationship (ECOSAR V2.0) was used [75,76]. The concentration of a compound that reduces 50% of fish and daphnia population is denoted as LC_50_, while EC_50_ represents a concentration of compound that allows for the normal growth of algae after 96 h. The values of EC_50_ and LC_50_ are given in mg L^−1^. In this contribution, and the classification of acute and chronic toxicity follows the Chinese hazard evaluation guidelines. According to this classification, there are four groups of compounds: not harmful (LC_50_, EC_50_ > 100, ChV > 10), harmful (10 < LC_50_, EC_50_ < 100, 1 < ChV < 10), toxic (1 < LC_50_, EC_50_ < 10, 0.1 < ChV < 1), and very toxic (LC_50_, EC_50_ < 1, ChV < 0.1).

## 4. Conclusions

Four new 4-hydroxycoumarin derivatives: **L1**, **L2**, **L3**, and **L4** were synthesized under mild conditions and were analyzed by IR and (^1^H and ^13^C) NMR spectroscopies. The crystal structure of **L2** was solved and it was shown that this compound crystallizes in the triclinic *P*-1 space group. The important structural parameter is the quasi-six membered ring (O–C–C–C–N–H) enclosed by the strong hydrogen bond. The crystallographic structure was reoptimized using several common functionals and, after the comparison between bond lengths and angles of experimental and theoretical structure, the B3LYP/6-311++G(d,p) level of theory was selected as being plausible. This was additionally proven by the comparison of the NMR spectra. The correlation coefficients between the experimental and theoretical chemical shifts were 0.978 (^1^H NMR) and 0.998 (^13^C NMR), while MAE values were 0.3 ppm and 1.4 ppm, respectively. The intramolecular interactions govern the stability of these compounds, especially within the quasi-six membered ring O–C–C–C–N–H. The obtained values of the interaction energies of these compounds are not significantly influenced by the number of OH groups, except for the OH group on the aliphatic chain. Based on the values of the HOMO-LUMO gap, **L4** and **L5** were selected as the most reactive. Cytotoxic activity was more pronounced for **L4** and **L5** towards the HCT-116 and HeLa cell lines. Excellent selectivity in comparison to normal healthy human fibroblast cells was observed for both compounds. The molecular docking results proved that the obtained compounds bind to the same amino acids (GLN92, GLU106, LEU198, THR199, THR200, and TRP209) of carbonic anhydrase IX (hCA-IX) as the native ligand, 5-Acetamido-1,3,4-thiadiazole-2-sulfonamide, AZA. The binding energies from the MD studies were significantly lower in the case of **L5** (−189.7 kJ mol^−1^) and **L4** (−184.9 kJ mol^−1^) than for the other compounds. This order follows the one obtained for the cytotoxic activity, thus proving the importance of MD for a better understanding of the biological activities at a molecular level. As far as stability is concerned, van der Waals interaction and hydrogen bonds are the main contributors to the stability of the obtained protein–ligand complexes. All of the compounds fulfill the Lipinski rules with toxicity within class IV values. Coumarin-neurotransmitter derivatives are inactive in terms of organ toxicity, carcinogenicity, mutagenicity, and cytotoxicity. The ecotoxicology assessment put these compounds into the category of the non-harmful substance when the fish and daphnia population are concerned in acute and chronic toxicity.

## Figures and Tables

**Table 1:** Crystal data and structure refinement of **L2**.

Compound	L2
Empirical formula	C_19_H_17_NO_5_
Formula weight	339.33
Temperature	95(2) K
Wavelength	1.54184 Å
Crystal system Space group	Triclinic *P*-1
Unit cell dimensions	*a* = 7.0726(2) Å → *α* = 99.978(2)° *b* = 9.8752(2) Å → *β* = 99.380(2)° *c* = 12.7283(3) Å → *γ* = 110.120(2)°
Volume	797.94(4) Å^3^
*Z*; density (calculated)	2; 1.412 g cm^−3^
Absorption coefficient	0.855 mm^−1^
*F*(000)	356
Crystal shape, color	prism, colorless
Crystal size	0.234 × 0.066 × 0.044 mm^3^
*θ* range for data collection	3.633–74.514°
Index ranges	−7 ≤ *h* ≤ 8, −12 ≤ *k* ≤ 12, −15 ≤ *l* ≤ 15
Reflections collected/independent	12478/3213 [*R*(int) = 0.0218]
Data/restraints/parameters	3213/0/248
Goodness-of-fit on *F*^2^	1.027
Final *R* indices [*I* >2σ(*I*)]	*R*1 = 0.0511, w*R*2 = 0.1265
*R* indices (all data)	*R*1 = 0.0563, w*R*2 = 0.1309
Largest diff. peak and hole	0.394; −0.486 e Å^−3^

**Table 2:** Hydrogen bonds for **L2** [Å and °].

D−H···A	d(D−H)	d(H···A)	d(D···A)	<(DHA)
C2′−H2’A···O2	0.98	1.90	2.709(2)	138.5
C7″−H7”···O41 i	0.95	2.38	3.235(4)	149.3
N1−H1N1···O3	0.92(3)	1.80(3)	2.5909(19)	142(2)
O5−H1O5···O2 ii	0.83(3)	1.92(4)	2.7236(19)	161(3)
O41−H1O4···O3 iii	0.92(4)	1.93(4)	2.797(2)	158(3)

Symmetry transformations used to generate equivalent atoms: (i): *x* + 1, *y*, *z*; (ii): *x* + 1, *y*, *z* + 1; (iii): −*x* + 1, −*y* + 1, −*z* + 1.

**Table 3:** Experimental and theoretical (at B3LYP-D3BJ/6-311++G(d,p), GIAO method) chemical shifts of **L2**. The enumeration follows Figure 1.

	δ(^1^H) (ppm)			δ(^13^C) (ppm)	
	Experimental	Theoretical		Experimental	Theoretical
C2′–H(3)	2.5	2.6	C2′	18.6	22.2
C1″–H(2)	3.4	3.8	C1″	51.6	53.8
O5–H	4.8	4.8	C2″	70.2	75.2
C2″–H	5.8	5.1	C3	96.1	98.1
C7″–H	6.7	7.1	C7″	115.0	113.2
C5″–H	6.7	7.1	C5″	115.0	114.4
C6–H	7.3	7.6	C8	116.2	116.1
C8–H	7.3	7.6	C10	120.4	119.9
C8″–H	7.3	7.6	C6	123.6	122.7
C7–H	7.6	7.8	C5	125.7	125.9
C4″–H	7.9	7.9	C4″	127.3	127.4
C5–H	9.4	8.5	C8″	127.3	129.3
N1–H	13.8	13.6	C7	132.7	133.9
MAE (ppm)	0.3		C3″	133.9	134.8
			C9	153.1	153.6
			C6″	156.8	156.5
			C2	162.0	159.9
			C1′	176.0	174.7
			C4	179.4	177.4
			MAE (ppm)	1.4	

**Table 4:** The interaction energies, electron density, and Laplacian of the intramolecular interactions.

	O–C–C–C–N–H			Aromatic Ring Substituents
Compound	Interaction Energy [kJ mol^−1^]	Electron Density [a.u.]	Laplacian [a.u.]	Interaction Energy [kJ mol^−1^]
**L1**	LP(O)→σ(N–H), 102	0.057	0.166	/
**L2**	LP(O)→σ(N–H), 86	0.052	0.158	/
**L3**	LP(O)→σ(N–H), 87	0.052	0.158	LP(O)→σ(O–H), 4
**L4**	LP(O)→σ(N–H), 93	0.055	0.162	LP(O)→σ(O–H), 17
**L5**	LP(O)→σ(N–H), 102	0.056	0.163	LP(O)→σ(O–H), 2

**Table 5:** The cytotoxic effects of **L1**, **L2**, **L3**, **L4**, and **L5** after 24 and 72 h of exposure.

IC₅₀, µM	HCT-116		HeLa		MDA-MB-231		MRC-5	
	24 h	72 h	24 h	72 h	24 h	72 h	24 h	72 h
**L1**	>500	170	>500	452	>500		>500	
**L2**		248		206				
**L3**		107		495				
**L4**		74		150				
**L5**		73		92				

**Table 6:** The important thermodynamic parameters for the best docking conformations of the investigated molecules with the hCA-IX receptor (PDB ID: 3IAI).

Conformations	ΔG_bind_ (kJ mol^−1^)	K_i_ (nM)	ΔG_vdw+hbond+desolv_ (kJ mol^−1^)	ΔG_elec_ (kJ mol^−1^)	ΔG_total_ (kJ mol^−1^)	ΔG_tor_ (kJ mol^−1^)	ΔG_unb_ (kJ mol^−1^)
hCA-IX-**AZA**	−37.8	237.83	−39.9	0.3	−2.3	2.8	0.0
hCA-IX-**L1**	−41.2	60.4	−38.8	−0.5	−6.7	4.8	0.0
hCA-IX-**L2**	−39.8	107.8	−39.1	−0.3	−6.1	5.7	0.0
hCA-IX-**L3**	−41.9	45.2	−39.5	−0.3	−8.7	6.7	0.0
hCA-IX-**L4**	−42.1	41.8	−41.0	0.2	−7.0	5.7	0.0
hCA-IX-**L5**	−43.7	21.9	−39.5	−1.0	−9.0	5.7	0.0

**Table 7:** Important thermodynamic parameters generated by the MM/PBSA protocol.

Complex	ΔE_elec_ (kJ mol^−1^)	ΔE_VDW_ (kJ mol^−1^)	ΔG_polar_ (kJ mol^−1^)	ΔG_nonpolar_ (kJ mol^−1^)	ΔG_binding_ (kJ mol^−1^)
hCA-IX-**L1**	−338.9 ± 27.7	−105.9 ± 14.4	393.6 ± 27.0	−17.2 ± 1.1	−68.4 ± 52.2
hCA-IX-**L2**	−333.4 ± 21.2	−116.0 ± 18.1	419.8 ± 29.3	−18.3 ± 1.1	−47.8 ± 39.8
hCA-IX-**L3**	−27.1 ± 27.0	−94.9 ±16.4	59.0 ± 17.9	−13.0 ± 1.2	−75.9 ± 24.3
hCA-IX-**L4**	−167.9 ± 1.4	−138.7 ± 1.2	141.1 ± 1.3	−19.3 ± 0.1	−184.9 ± 1.3
hCA-IX-**L5**	−351.0 ± 26.6	−135.4 ± 16.5	316.1 ± 26.6	−19.5 ± 1.2	−189.7 ± 27.1

**Table 8:** Toxicity evaluation of the coumarin-neurotransmitter derivatives (mg L^−1^).

Compound	LC_50_ (Fish, 96 h)	LC_50_ (Daphnia, 48 h)	EC_50_ (Green Algae, 96 h)	ChV (Fish, Chronic)	ChV (Daphnia, Chronic)	ChV (Green Algae, Chronic)
**L1**	424	96.3	35.3	36.3	8.2	53.4
**L2**	5080	576	399	372	37.6	419
**L3**	2150	66,400	109	2000	30,600	8.78
**L4**	611	128	50.6	51.4	10.6	73.3
**L5**	249	4170	31.5	184	1750	3.18

## Data Availability

Data are contained within the article and supplementary material.

## Supplementary Materials

CCDC 2126116 contains the supplementary crystallographic data for **L2**. These data can be obtained free of charge via http://www.ccdc.cam.ac.uk/conts/retrieving.html (accessed 1 January 2022), or from the Cambridge Crystallographic Data Centre, 12 Union Road, Cambridge CB2 1EZ, UK; fax: (+44) 1223-336-033; or e-mail: deposit@ccdc.cam.ac.uk. The following supporting information can be downloaded at: https://www.mdpi.com/article/10.3390/ijms23021001/s1. Reference [77] is cited in the supplementary materials.
