# Supplementary material for: Synthesis, Crystallographic, Quantum Chemical, Antitumor, and Molecular Docking/Dynamic Studies of 4-Hydroxycoumarin-Neurotransmitter Derivatives
Source: Int J Mol Sci. 2022 Jan 17;23(2):1001. doi: 10.3390/ijms23021001 (PMC8780855; doi:10.3390/ijms23021001)
Supplement: Supplementary file 1 [file ijms-23-01001-s001.zip › ijms-1522667-supplementary.pdf]

Supplementary Information for:

Synthesis, Crystallographic, Quantum Chemical, Antitumor, and Molecular Docking/Dynamic Studies of 4-Hydroxycoumarin-Neurotransmitter Derivatives

Dušan S. Dimić [1,*], Goran N. Kaluđerović [2], Edina H. Avdović [3], Dejan A. Milenković [3], Marko N. Živanović [3], Ivan Potočňák [4], Erika Samoľová [5], Milena S. Dimitrijević [6], Luciano Saso [7], Zoran S. Marković [3] and Jasmina M. Dimitrić Marković [1]

[1] Faculty of Physical Chemistry, University of Belgrade, Studentski trg 12-16, 11000 Belgrade, Serbia; markovich@ffh.bg.ac.rs

[2] Department of Engineering and Natural Sciences, University of Applied Sciences Merseburg, Eberhard-Leibnitz-Straße 2, DE-06217 Merseburg, Germany; goran.kaluderovic@hs-merseburg.de

[3] Department of Science, Institute for Information Technologies, University of Kragujevac, Jovana Cvijića bb, 34000 Kragujevac, Serbia; edina.avdovic@pmf.kg.ac.rs (E.H.A.); deki82@kg.ac.rs (D.A.M.); marko.zivanovic@uni.kg.ac.rs (M.N.Ž.); zmarkovic@uni.kg.ac.rs (Z.S.M.)

[4] Institute of Chemistry, P. J. Šafárik University in Košice, Moyzesova 11, 04154 Košice, Slovakia; ivan.potocnak@upjs.sk

[5] Institute of Physics of the Czech Academy of Sciences, Na Slovance 2, 182 21 Prague 8, Czech Republic; samolova@fzu.cz

[6] Department of Life Sciences, Institute for Multidisciplinary Research, University of Belgrade, Kneza Višeslava 1, 11030 Belgrade, Serbia; milena.dimitrijevic@imsi.bg.ac.rs

[7] Department of Physiology and Pharmacology "Vittorio Erspamer", Sapienza University of Rome, P.le Aldo Moro 5, 00185 Rome, Italy; luciano.saso@uniroma1.it

* Correspondence: ddimic@ffh.bg.ac.rs

1. Fukui functions

Fukui functions are very useful parameters when it comes to the investigation of the possible electrophilic, nucleophilic, and radical attack sites. These parameters can also be used for the prediction of the binding sites for interactions with various amino acids in proteins. Fukui functions describe the change in total electron density when anions and cations are formed. Their evaluation depends on the chosen methodology, and several ways for their calculation are found in the literature [64,77]. Condensed Fukui functions (CFF) can be determined for the specific atoms in the following way:

$$f_A^+ = q_N^A - q_{N+1}^A \quad (S1)$$

$$f_A^- = q_{N-1}^A - q_N^A \quad (S2)$$

$$f_A^0 = [q_{N-1}^A - q_{N+1}^A]/2 \quad (S3)$$

To obtain CFFs, the charges on atom A have to be determined in the neutral, anionic, and cationic species. When CFF values are compared, a higher value represents a more reactive site [65]. The CFFs were calculated in the Multiwfn program package [66].

2. Molecular Docking—Binding free energy

The AutoDock program calculates the free energy of binding values according to the following equation, Equation (S4):

$$\Delta\mathbf{G}_{bind} = \Delta\mathbf{G}_{vdw+hbond+desolv} + \Delta\mathbf{G}_{elec} + \Delta\mathbf{G}_{total} + \Delta\mathbf{G}_{tor} - \Delta\mathbf{G}_{unb} \quad (S4)$$

where $\Delta\mathbf{G}_{bind}$ is the estimated free energy of binding, and $\Delta\mathbf{G}_{vdw+hbond+desolv}$ denotes the sum of the energies of dispersion and repulsion ($\Delta\mathbf{G}_{vdw}$), hydrogen bond ($\Delta\mathbf{G}_{hbond}$), and desolvation ($\mathbf{\Delta G}_{desolv}$). $\Delta\mathbf{G}_{total}$ represents the final total internal energy, $\Delta\mathbf{G}_{tor}$ is torsional free energy, $\Delta\mathbf{G}_{unb}$ is unbound system's energy, and $\Delta\mathbf{G}_{elec}$ is electrostatic energy.

3. Molecular Dynamics—Binding free energy

The free energy of the binding of the investigated complex was determined by the following equation (Equation (S5)).

$$\Delta\mathbf{G}_{bind} = \Delta\mathbf{G}_{complex} - (\Delta\mathbf{G}_{protein} + \Delta\mathbf{G}_{ligand}) \quad (S5)$$

where $\Delta\mathbf{G}_{bind}$ is the binding free energy of hCA-IX with investigated compounds, $\Delta\mathbf{G}_{complex}$ is the total free energy hCA-IX with investigated compounds, and $\Delta\mathbf{G}_{protein}$ and $\Delta\mathbf{G}_{ligand}$ are total free energies of hCA-IX and investigated ligands in a solvent, respectively. The free energy of the individual molecules was estimated according to Equation (S6):

$$\Delta\mathbf{G}_{molecule} = \Delta\mathbf{E}_{MM} - TS + \Delta\mathbf{G}_{solvatation} \quad (S6)$$

where $\Delta\mathbf{G}_{molecule}$ is the free energy of protein, ligand, or protein-ligand complex, and $\mathbf{E}_{MM}$ is the average molecular mechanics (MM) potential energy in the vacuum. TS denotes the entropic contribution to the free energy, while T and S represent the temperature and entropy, respectively. On the other hand, $\Delta\mathbf{G}_{solvatation}$ represents the free energy solvation to transfer a solute from a vacuum to a solvent. $\Delta\mathbf{E}_{MM}$ (Equation (S7)) is expressed as follows:

$$\Delta\mathbf{E}_{MM} = \Delta\mathbf{E}_{bonded} + \Delta\mathbf{E}_{nonbonded} = \Delta\mathbf{E}_{bonded} + \Delta\mathbf{E}_{vdw} + \Delta\ \mathbf{E}_{elec} \quad (S7)$$

$\Delta\mathbf{E}_{bonded}$ is the bonded interactions that contribute to the bond, angle, dihedral, and improper interactions, while $\Delta\mathbf{E}_{nonbonded}$ denotes nonbonded interaction involving the van der Waals electrostatic

energy terms. $\Delta\mathbf{E}_{bonded}$ is always taken as zero. $\Delta\mathbf{G}_{solvatation}$ is an energy term calculated in an implicit solvent as shown in Equation (S8):

$$\Delta\mathbf{G}_{solvatation} = \Delta\mathbf{G}_{polar} + \Delta\mathbf{G}_{nonpolar} \tag{S8}$$

$\Delta\mathbf{G}_{polar}$ refers to the solvation free energy of the electrostatic interactions calculated by solving the Poisson−Boltzmann (PB) equation, and $\Delta\mathbf{G}_{nonpolar}$ represents non-electrostatic interactions to the solvation free energy, which refers to the solvent-accessible surface area (SASA) model. The obtained results of the average binding energy (kJ mol^{-1}) calculations can offer better insight into the interactions of the above-mentioned complexes.

Table S1: HPLC data for compound 4-hydroxycoumarin.

	Retention time (min)	Area	Peak height	%
1	3.30	8926	1074	0.21
**2**	**9.36**	**4132824**	**914754**	**98.26**
3	11.22	3793	316	0.09
4	13.75	494	121	0.01
5	14.99	2289	511	0.05
6	16.65	53207	18729	1.27
7	17.77	4542	307	0.11
Wavelentght: 280 nm; Duration 20 min; Volume: 20µL				

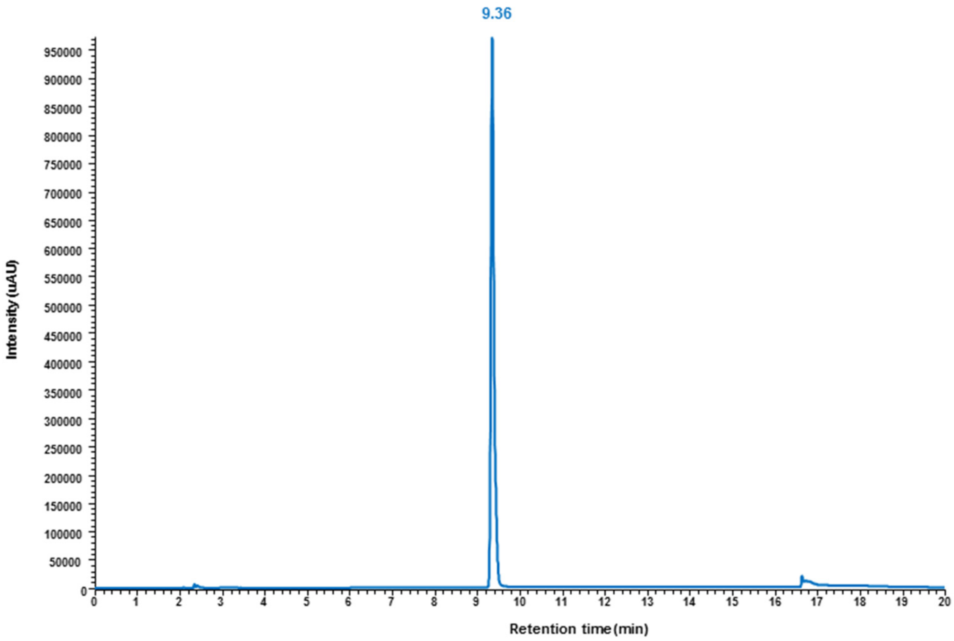

Figure S1: HPLC chromatogram of 4-hydroxycoumarin.

Table S2: HPLC data for compound **3-acetyl-4-hydroxycoumarin**.

	Retention time (min)	Area	Peak height	%
1	3.45	5691	977	0.24
2	9.36	3748	873	0.21
3	11.48	587	174	0.04
**4**	**12.33**	**2501926**	**386780**	**94.58**
5	13.44	800	187	0.05
6	15.02	19635	1651	0.40
7	16.65	53699	18996	4.65
8	17.77	3978	290	0.07
Wavelentght: 280 nm; Duration 20 min; Volume: 20µL				

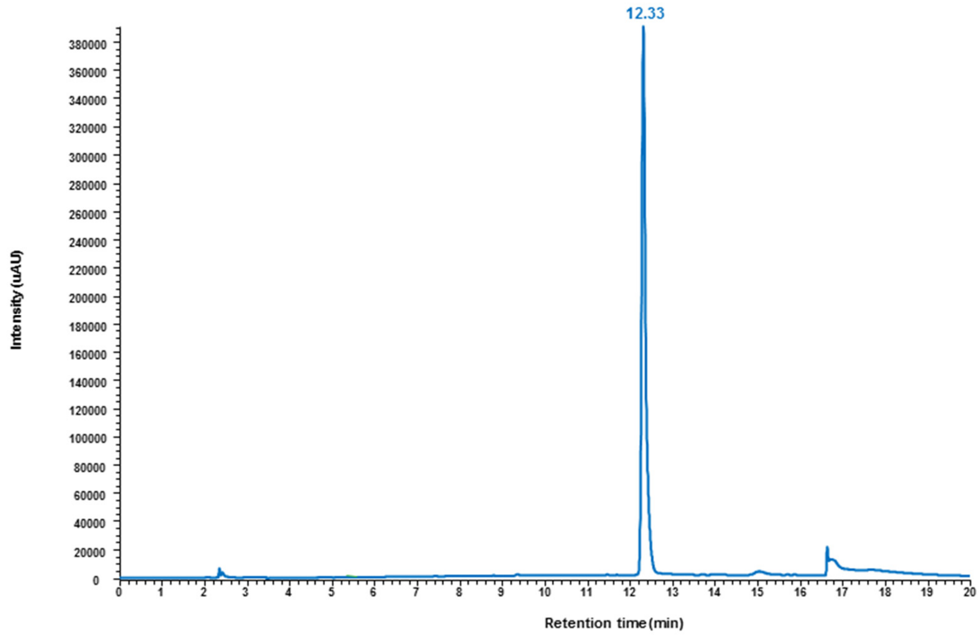

Figure S2: HPLC chromatogram of compound **3-acetyl-4-hydroxycoumarin**.

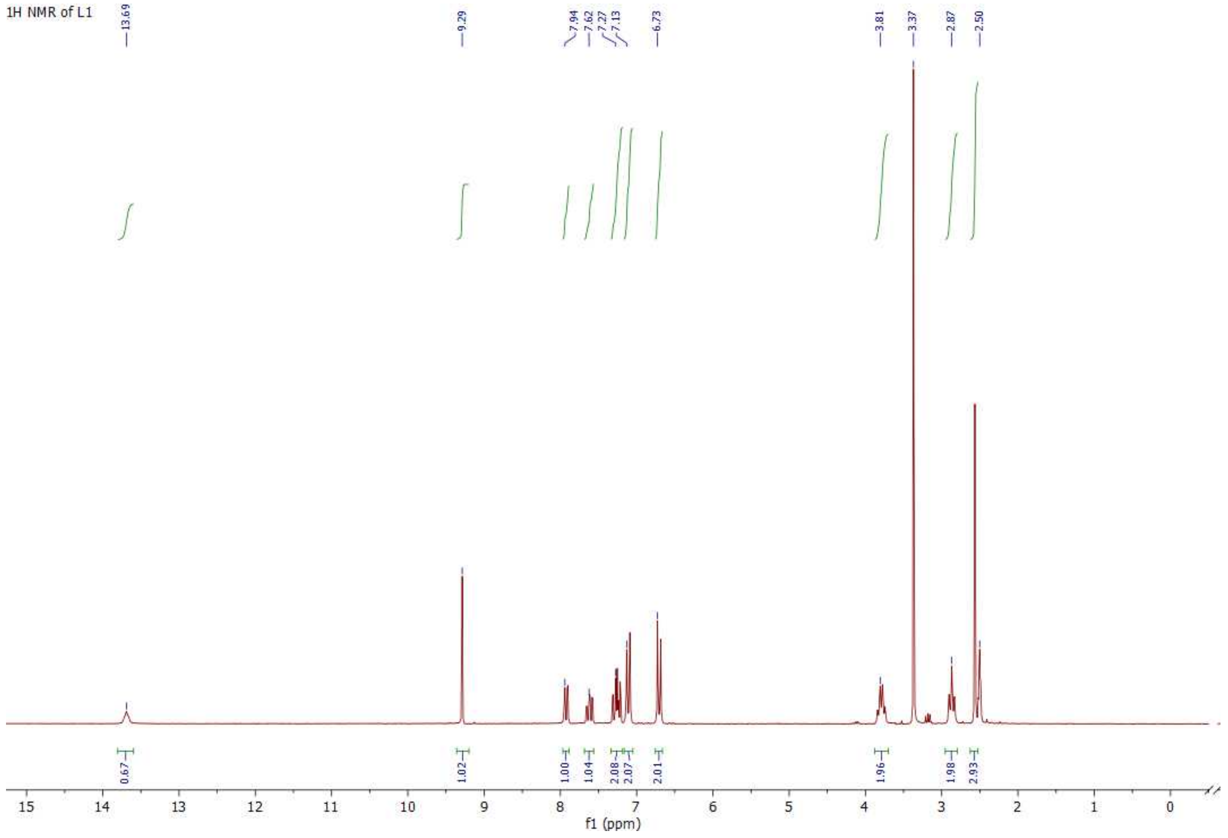

Figure S3. The ^{1}H spectrum of L1.

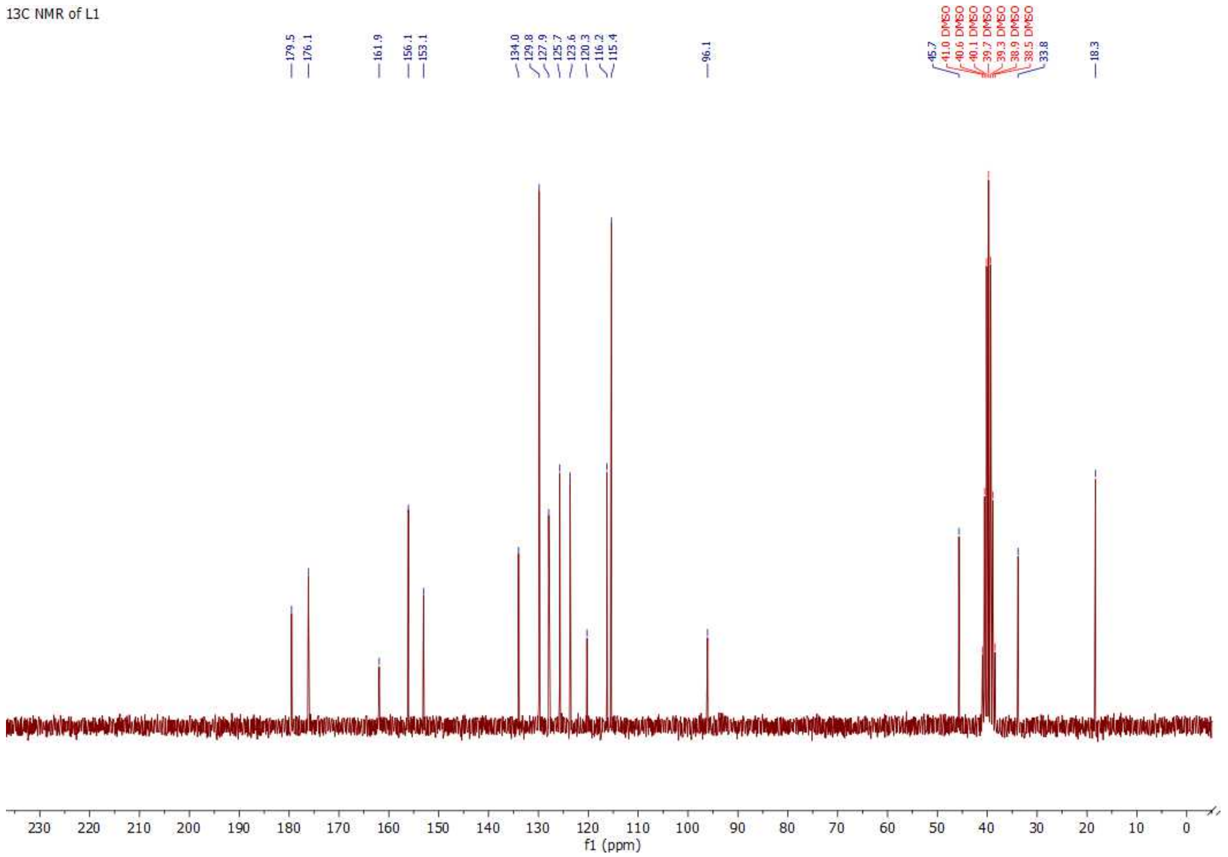

Figure S4. The ^{13}C spectrum of L1.

Table S3: HPLC data for compound **L1**.

	Retention time (min)	Area	Peak height	%
1	3.44	6264	1139	0.25
**2**	**11.39**	**2452787**	**471063**	**99.34**
3	12.32	8384	54357	0.34
4	16.65	1569	19055	0.06
Wavelentght: 280 nm; Duration 20 min; Volume: 20µL				

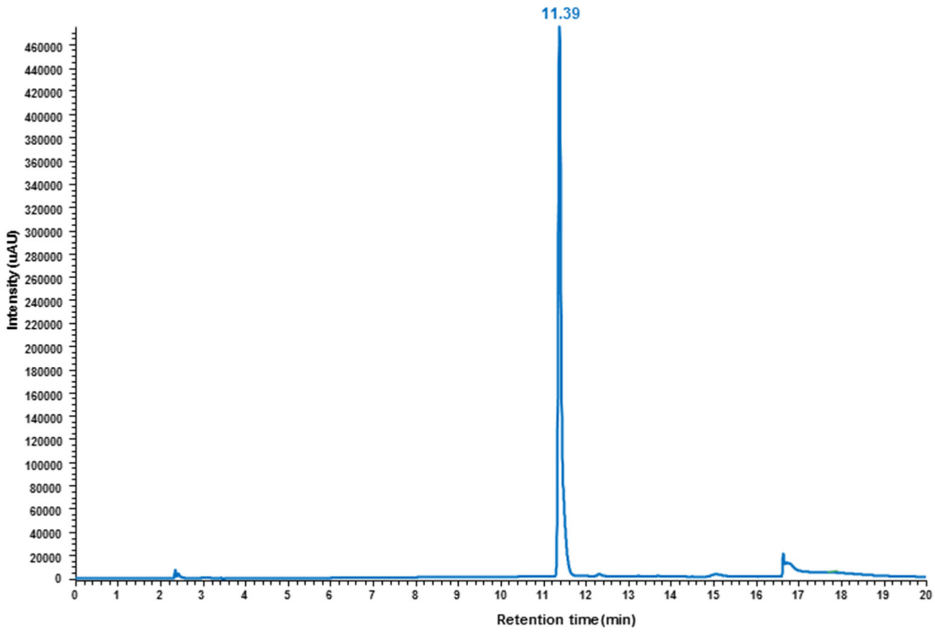

Figure S5: HPLC chromatogram of compound **L1**.

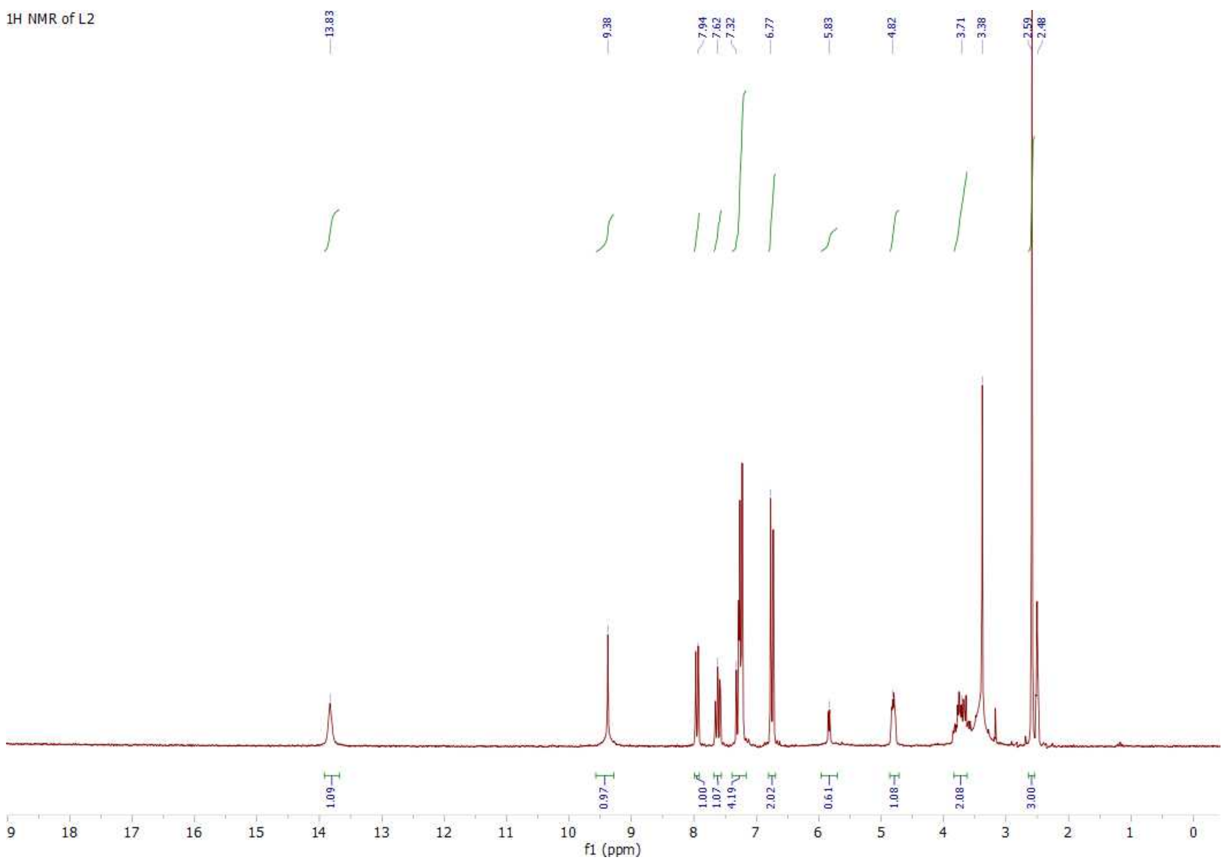

Figure S6. The ^{1}H spectrum of L2.

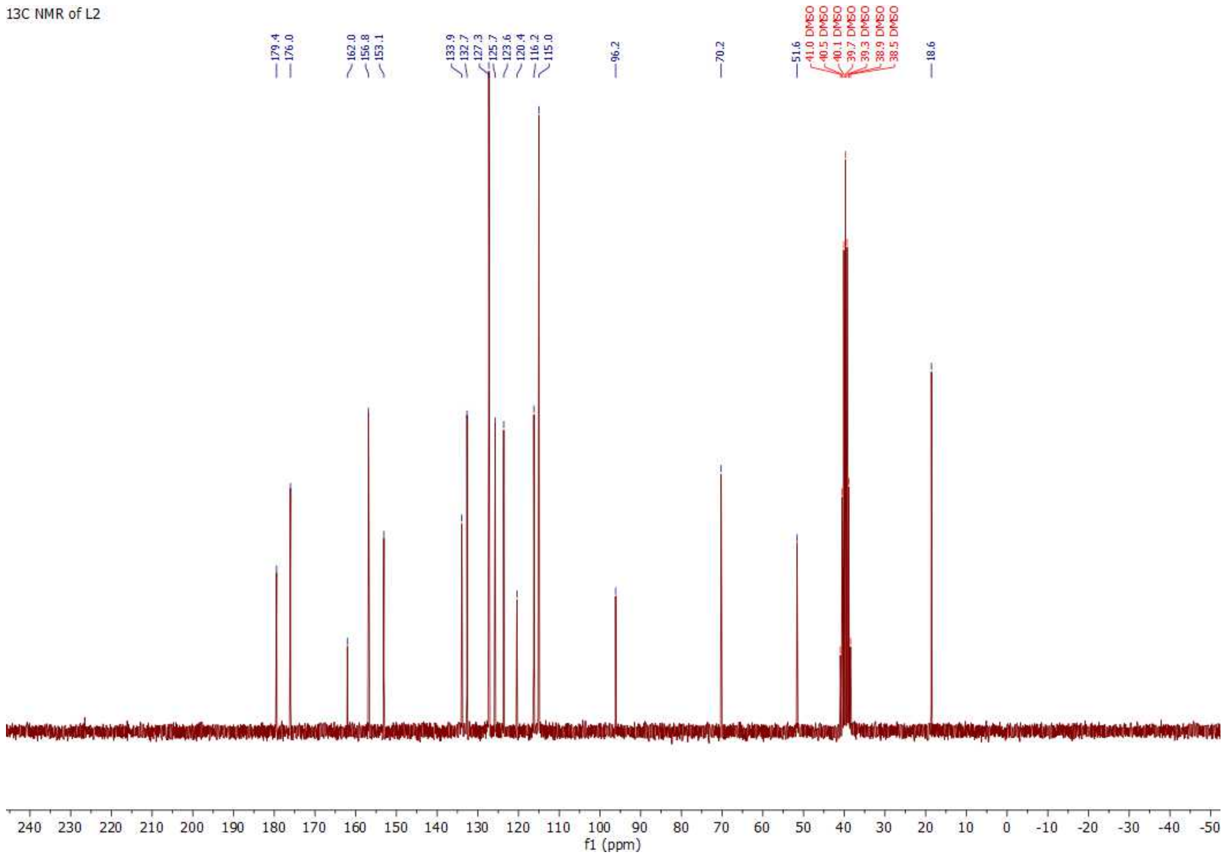

Figure S7. The ^{13}C spectrum of L2.

Table S4: HPLC data for compound **L2**.

	Retention time (min)	Area	Peak height	%
1	3.44	5550	998	0.20
2	9.56	1210	316	0.04
**3**	**10.03**	**2744256**	**528444**	**97.70**
4	12.33	3986	654	0.14
5	16.65	53979	19005	1.92
Wavelentght: 280 nm; Duration 20 min; Volume: 20µL				

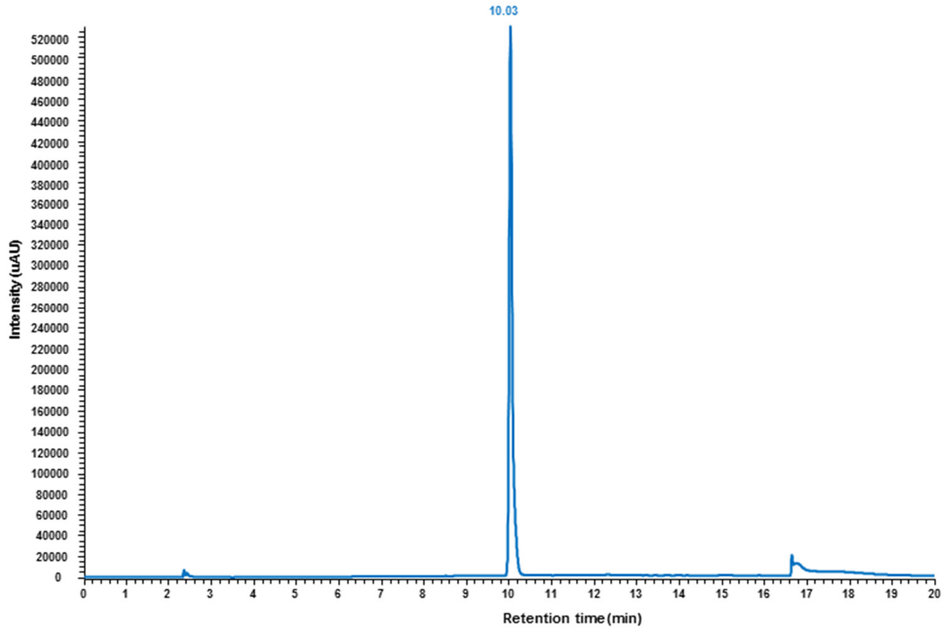

Figure S8: HPLC chromatogram of compound **L2**.

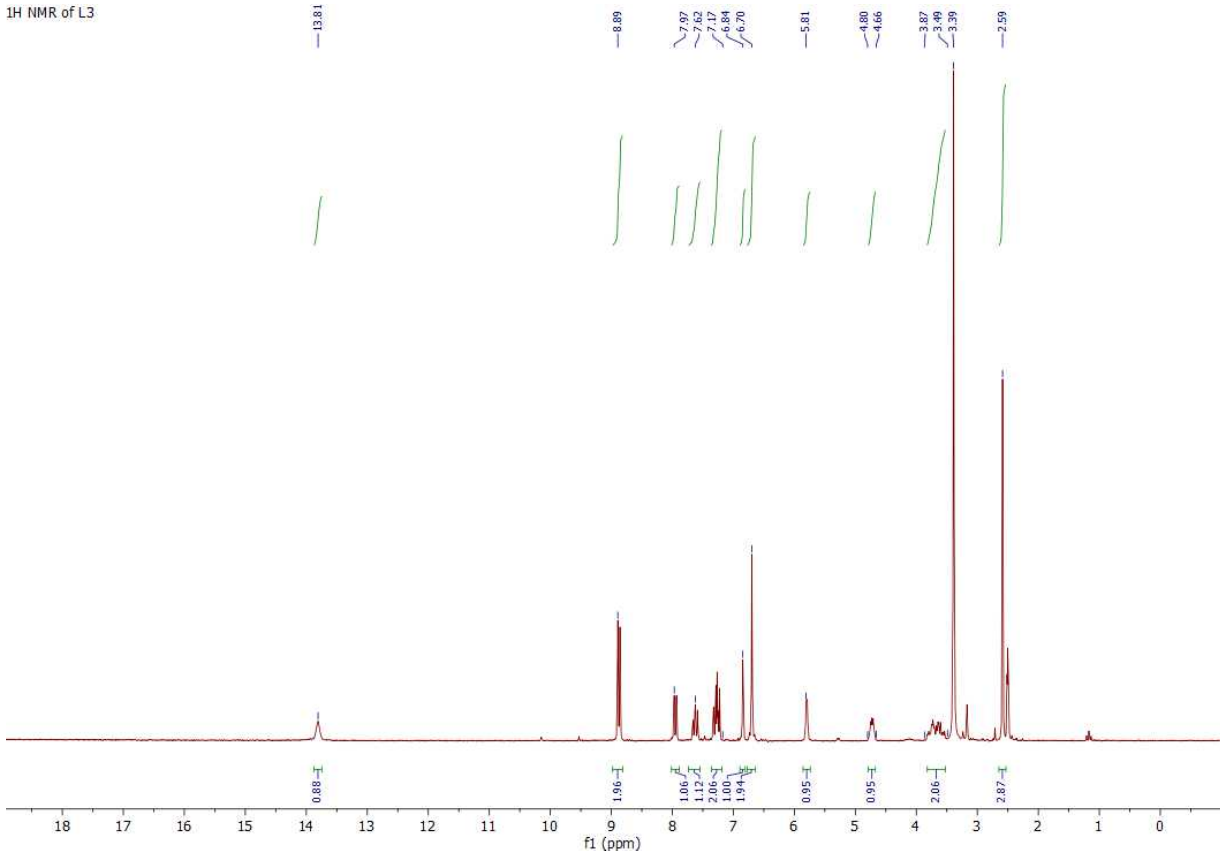

Figure S9. The ^1H spectrum of L3.

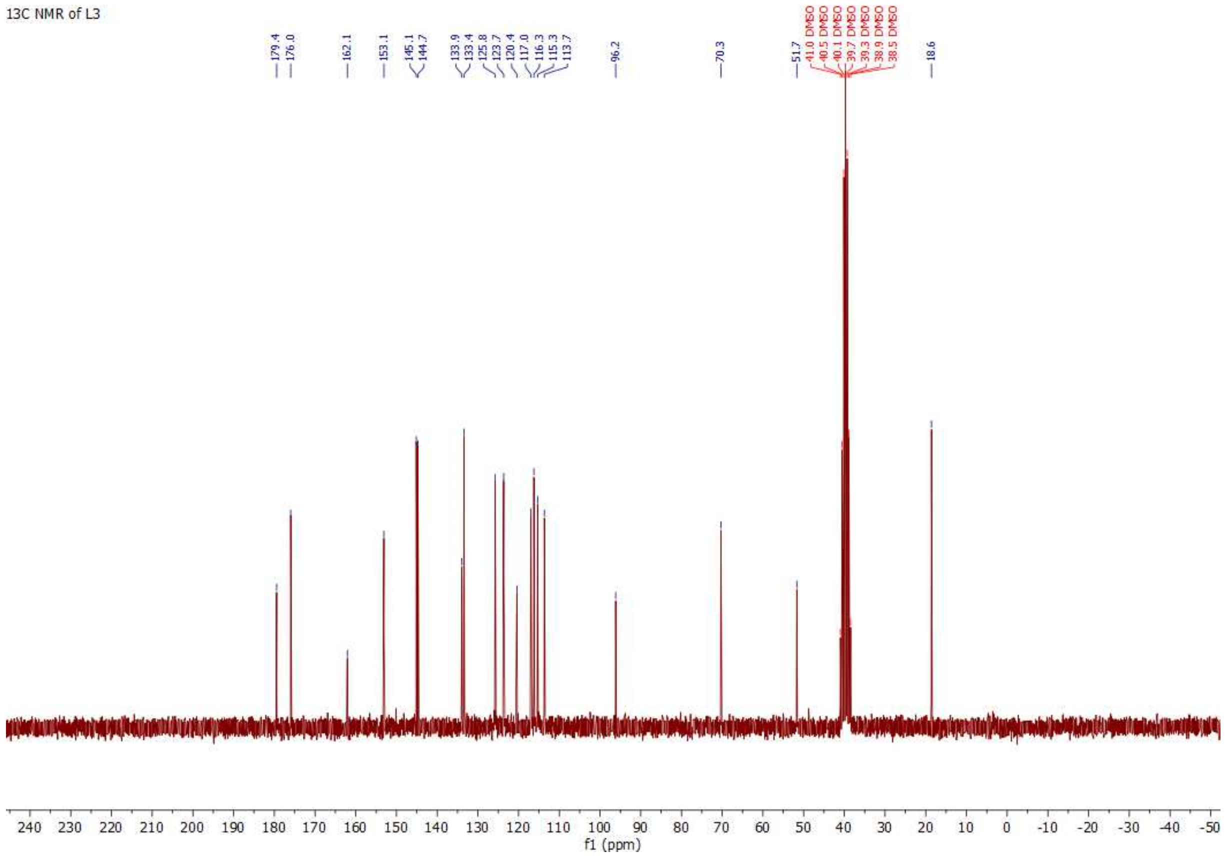

Figure S10. The ^{13}C spectrum of L3.

Table S5: HPLC data for compound **L3**.

	Retention time (min)	Area	Peak height	%
1	3.44	5278	1027	0.13
2	6.62	1598	385	0.04
3	10.11	52872	13096	1.28
**4**	**11.55**	**3965357**	**959713**	**96.25**
5	12.24	41573	8727	1.01
6	16.65	48500	18246	1.18
7	17.71	4684	293	0.11
Wavelentght: 280 nm; Duration 20 min; Volume: 20µL				

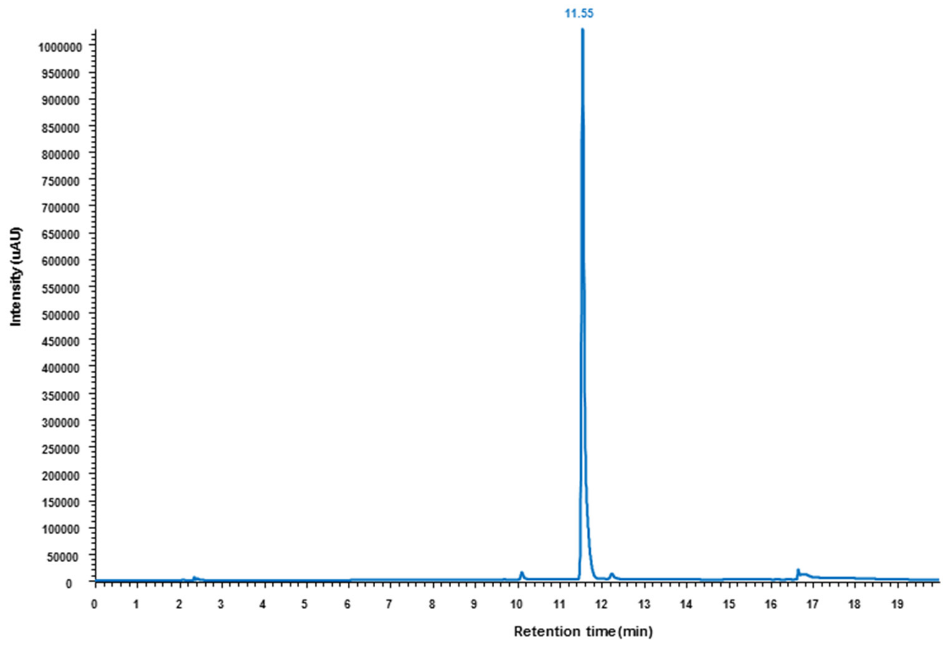

Figure S11: HPLC chromatogram of compound **L3**.

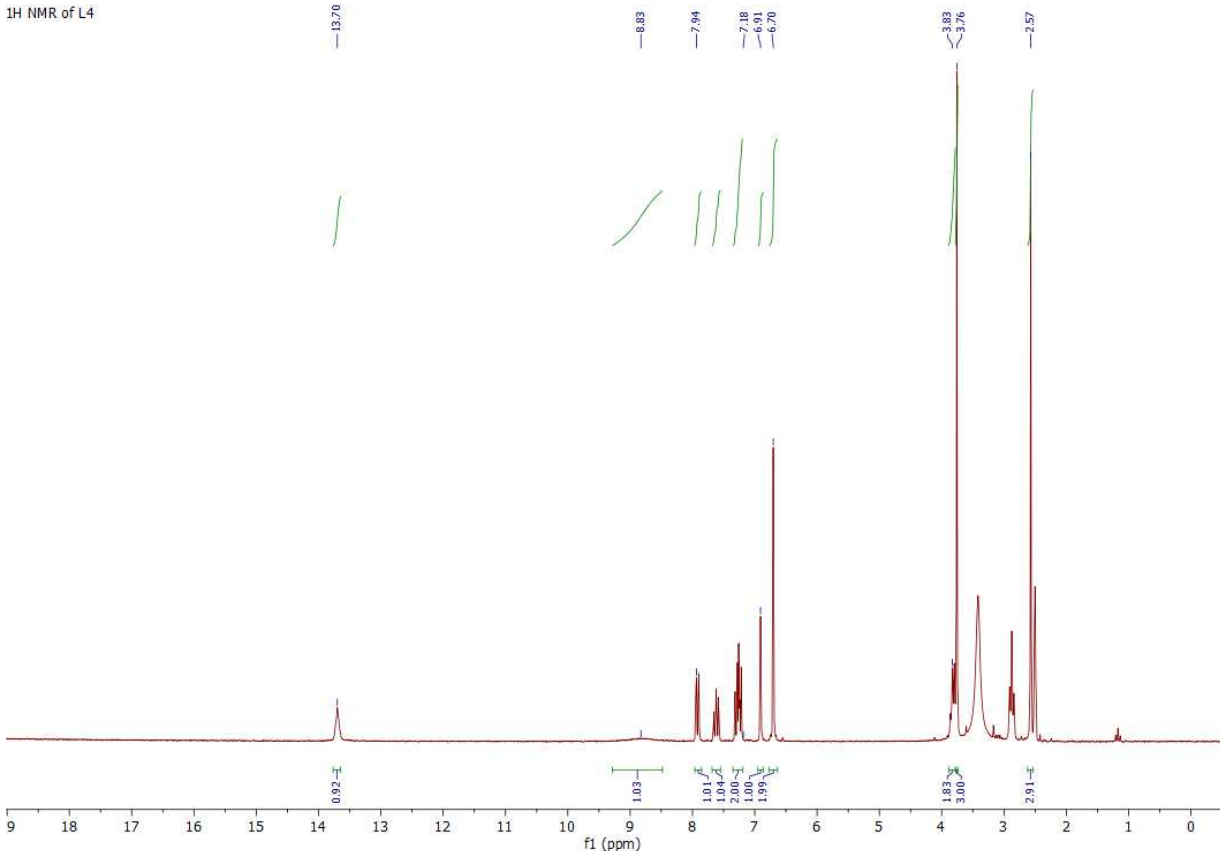

Figure S12. The ^{1}H spectrum of **L4**.

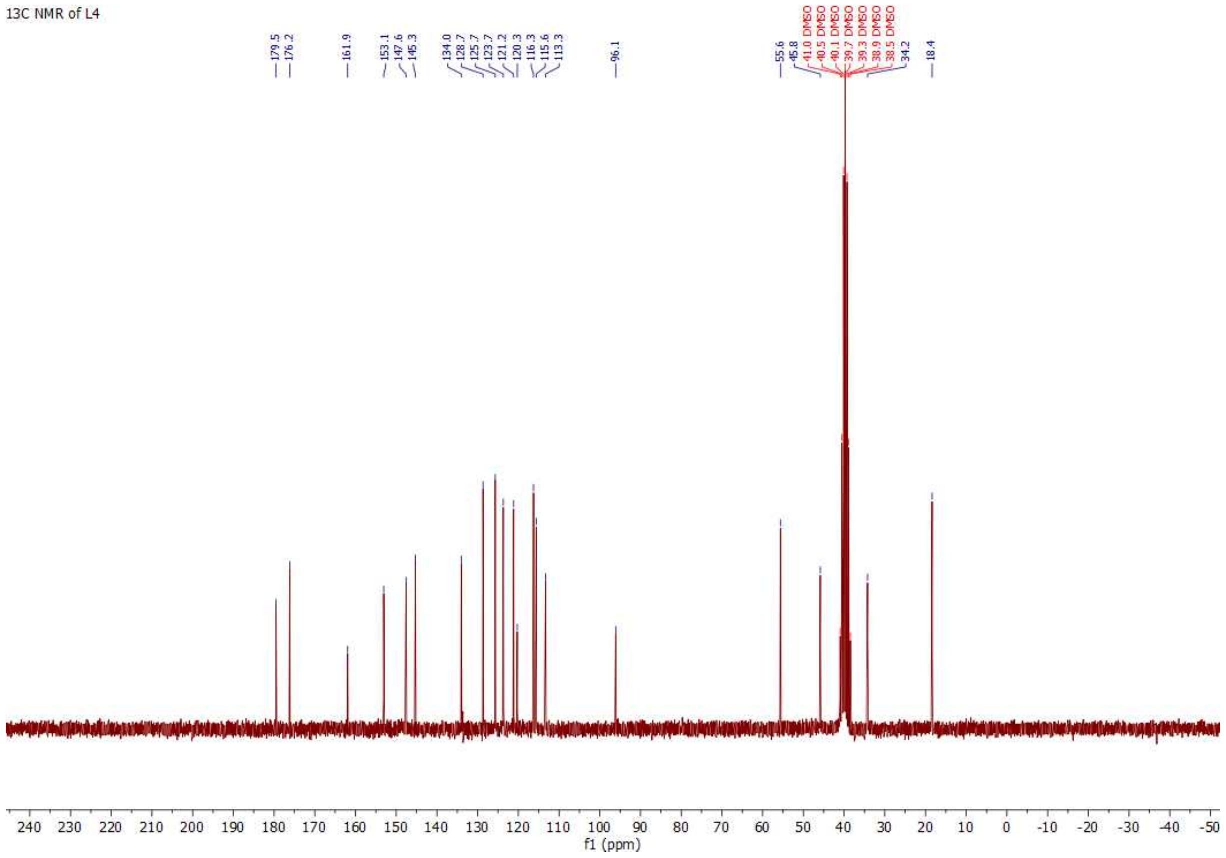

Figure S13. The ^{13}C spectrum of **L4**.

Table S6: HPLC data for compound **L4**.

	Retention time (min)	Area	Peak height	%
1	3.44	6174	1035	0.21
2	7.77	365	113	0.01
3	9.93	17585	4309	0.59
**4**	**11.38**	**2734788**	**497100**	**91.84**
5	12.24	168786	38723	5.67
6	16.65	45619	17835	1.53
7	17.75	4560	288	0.15
Wavelentght: 280 nm; Duration 20 min; Volume: 20µL				

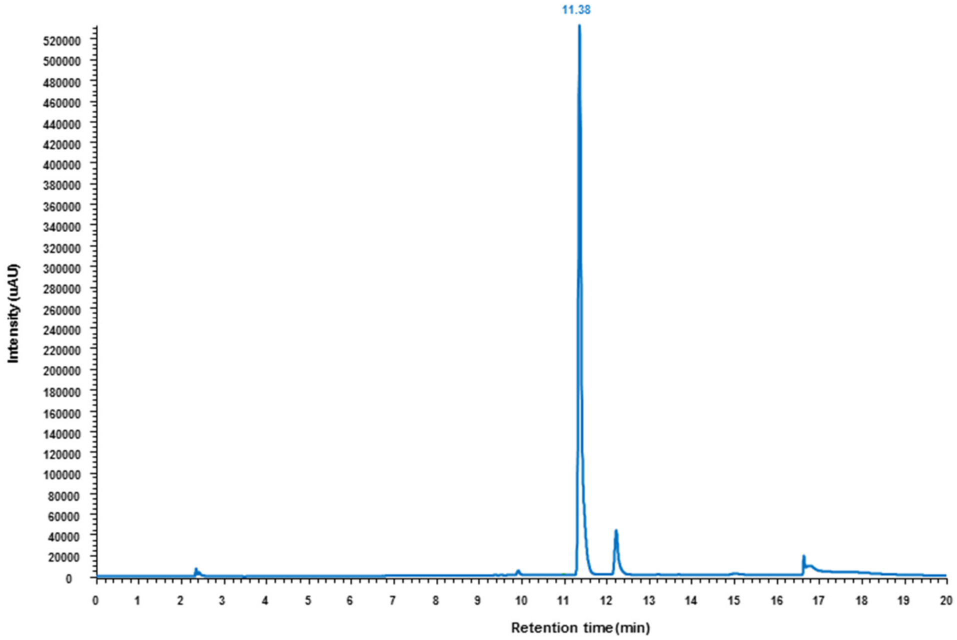

Figure S14: HPLC chromatogram of compound **L4**.

Table S7: HPLC data for compound **L5**.

	Retention time (min)	Area	Peak height	%
1	3.44	6623	1084	0.23
**2**	**10.59**	**2807145**	**546626**	**97.37**
3	12.32	8877	1683	0.31
4	13.71	3868	805	0.13
5	16.65	51775	18657	1.80
6	17.79	4544	310	0.16
Wavelentght: 280 nm; Duration 20 min; Volume: 20µL				

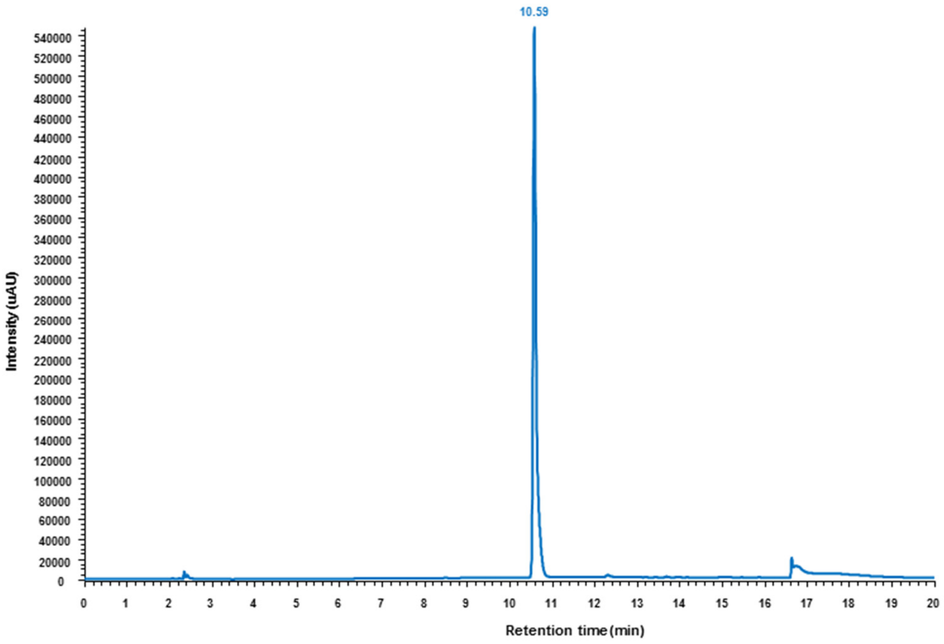

Figure S15: HPLC chromatogram of compound **L5**.

Table S8. Bond lengths in crystallographic and optimized structures (given functionals in conjunction with 6-311++G(d,p) basis set) of **L2** [Å].

		APFD	B3LZP-D3	M05-2X	M06-2X
O1-C2	1.375 (2)	1.388	1.393	1.381	1.380
C2-O2	1.223 (2)	1.208	1.210	1.204	1.204
C2-C3	1.439 (2)	1.455	1.457	1.455	1.459
C3-C4	1.441 (2)	1.447	1.451	1.446	1.449
C4-O3	1.254 (2)	1.245	1.248	1.239	1.237
C4-C10	1.472 (2)	1.466	1.471	1.472	1.476
C10-C5	1.403 (2)	1.398	1.402	1.397	1.398
C10-C9	1.385 (2)	1.393	1.394	1.386	1.389
C5-C6	1.381 (2)	1.383	1.385	1.381	1.383
C6-C7	1.395 (2)	1.400	1.401	1.398	1.399
C7-C8	1.380 (2)	1.385	1.387	1.383	1.384
C8-C9	1.389 (2)	1.394	1.395	1.392	1.394
C9-O1	1.375(2)	1.356	1.363	1.360	1.359
C3-C1'	1.441 (2)	1.424	1.426	1.421	1.421
C1'-C2'	1.495 (2)	1.496	1.501	1.499	1.501
C1'-N1	1.305 (2)	1.321	1.326	1.319	1.323
N1-C1''	1.467 (2)	1.447	1.456	1.455	1.454
C1''-C2''	1.457 (3)	1.523	1.527	1.521	1.524
C2''-C3''	1.517 (3)	1.510	1.513	1.510	1.512
C3''-C4''	1.375 (2)	1.393	1.396	1.391	1.393
C4''-C5''	1.392 (2)	1.389	1.391	1.388	1.389
C5''-C6''	1.372 (2)	1.396	1.396	1.392	1.393
C6''-O5	1.365 (2)	1.359	1.366	1.361	1.360
C6''-C7''	1.385 (2)	1.394	1.395	1.390	1.393
C7''-C8''	1.377 (2)	1.387	1.390	1.386	1.387
C8''-C3''	1.369 (2)	1.395	1.397	1.393	1.395

Table S9. Bond angles in crystallographic and optimized structures (given functionals in conjunction with 6-311++G(d,p) basis set) of **L2** [°].

		APFD	B3LZP-D3	M05-2X	M06-2X
O1-C2-O2	113.0	115.2	115.0	115.4	115.3
O1-C2-C3	119.1	117.8	117.7	117.7	117.8
O2-C2-C3	127.8	127.0	127.3	126.9	126.8
C2-C3-C1'	119.2	119.1	119.0	118.8	118.9
C2-C3-C4	120.1	120.7	120.7	120.9	120.7
C3-C4-O3	123.4	123.8	123.7	124.2	124.2
C3-C4-C10	117.2	116.8	116.8	116.5	116.3
O3-C4-C10	119.4	119.4	119.5	119.4	119.4

C4-C10-C5	122.4	121.2	121.3	121.0	121.0
C4-C10-C9	119.5	119.6	119.7	119.7	119.7
C10-C5-C6	120.4	120.4	120.5	120.3	120.3
C5-C6-C7	119.8	119.6	119.7	119.6	119.6
C6-C7-C8	121.1	120.8	120.7	120.8	120.8
C7-C8-C9	118.1	119.0	119.0	118.9	119.0
C8-C9-C10	122.6	121.0	121.1	121.1	121.0
C8-C9-O1	115.9	116.8	116.9	116.8	116.8
C3-C1'-C2'	122.3	122.9	122.7	122.6	122.6
C3-C1'-N1	119.6	118.6	119.0	119.4	119.4
C2'-C1'-N1	118.1	118.5	118.3	118.0	117.9
C1'-N1-C1''	126.8	127.5	127.4	127.1	127.3
N1-C1''-C2''	111.9	109.4	109.6	108.8	108.9
C1''-C2''-C3''	110.8	110.4	111.0	110.4	110.6
C2''-C3''-C4''	122.8	120.4	120.7	120.3	120.3
C2''-C3''-C8''	118.9	120.8	120.7	120.8	120.9
C3''-C4''-C5''	121.6	120.8	120.8	120.6	120.7
C4''-C5''-C6''	119.2	119.9	119.9	119.8	119.8
C5''-C6''-O5	123.0	123.1	122.7	122.4	122.6
C5''-C6''-C7''	119.4	119.8	119.9	120.2	120.1
O5-C6''-C7''	117.6	117.1	117.4	117.4	117.3
C6''-C7''-C8''	120.3	119.7	119.5	119.5	119.5
C7''-C8''-C3''	121.1	121.1	121.2	121.0	121.1

Table S10. Values of R and MAE for the optimized structures at various level of theory.

		APFD	B3LZP-D3	M05-2X	M06-2X
Bond lengths	**R**	0.967	0.970	0.970	0.970
	**MAE** [Å]	0.012	0.013	0.011	0.013
Bond angles	**R**	0.960	0.964	0.954	0.954
	**MAE** [°]	0.77	0.72	0.82	0.81

Table S11. Reactivity parameters of the compounds **L1**-**L5**.

Compound	HOMO energy [eV]	HOMO-LUMO gap [eV]	Dipole moment [D]
**L1**	-6.41	4.41	3.16
**L2**	-6.44	4.53	5.70
**L3**	-6.44	4.49	4.76
**L4**	-6.03	4.06	5.60
**L5**	-6.16	4.15	5.35

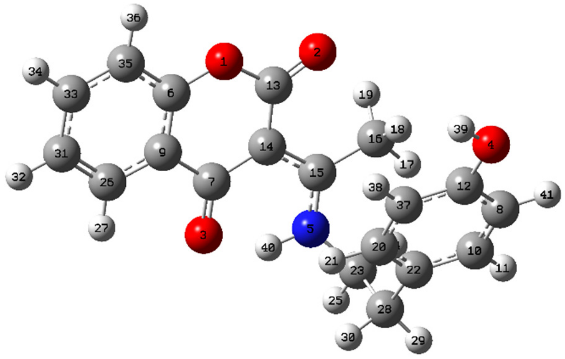

Figure S16. The atom numbering scheme for **L1**.

Table S12. CFFs for the specific atoms of **L1**.

Atom	f-	f+	f0
1(O)	0.022	0.020	0.021
2(O)	**0.077**	0.042	**0.060**
3(O)	0.033	**0.074**	**0.054**
4(O)	**0.057**	0.014	0.036
5(N)	0.032	0.051	0.041
6(C)	0.022	0.028	0.025
7(C)	0.013	**0.069**	0.041
8(C)	0.039	0.016	0.027
9(C)	0.016	0.023	0.020
10(C)	0.025	0.009	0.017
11(H)	0.021	0.014	0.017
12(C)	0.039	0.010	0.024
13(C)	0.024	0.018	0.021
14(C)	**0.062**	0.016	0.039
15(C)	0.010	**0.078**	0.044
16(C)	0.007	0.020	0.013
17(H)	0.011	0.019	0.015
18(H)	0.002	0.021	0.012
19(H)	0.014	0.024	0.019
20(C)	0.020	-0.008	0.006
21(H)	0.012	-0.005	0.004
22(C)	0.040	-0.005	0.018
23(C)	0.017	0.014	0.015
24(H)	0.013	0.018	0.016
25(H)	0.023	0.023	0.023
26(C)	0.017	0.044	0.031
27(H)	0.015	0.027	0.021
28(C)	0.016	0.011	0.013
29(H)	0.023	0.029	0.026

30(H)	0.015	0.009	0.012
31(C)	0.042	0.035	0.038
32(H)	0.023	0.027	0.025
33(C)	0.039	0.067	**0.053**
34(H)	0.023	0.038	0.030
35(C)	0.021	0.038	0.029
36(H)	0.017	0.027	0.022
37(C)	0.024	0.002	0.013
38(H)	0.016	0.004	0.010
39(H)	0.022	0.007	0.015
40(H)	0.011	0.016	0.014
41(H)	0.025	0.015	0.020

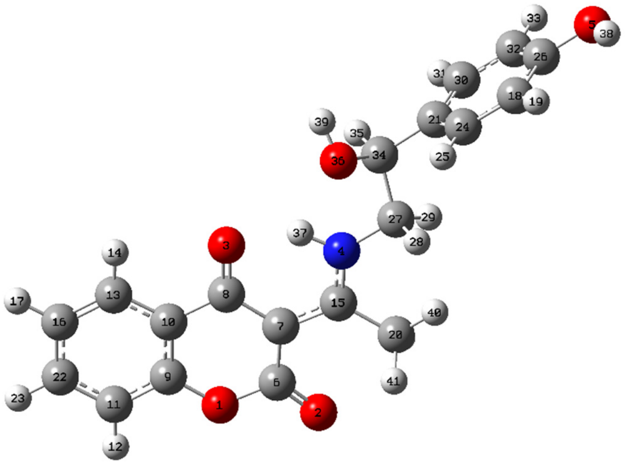

Figure S17. The atom numbering scheme for **L2**.

Table S13. CFFs for the specific atoms of **L2**.

Atom	f-	f+	f0
1(O)	0.027	0.017	0.022
2(O)	**0.088**	0.036	**0.062**
3(O)	0.030	**0.059**	0.044
4(N)	0.041	0.039	0.040
5(O)	**0.051**	0.021	0.036
6(C)	0.027	0.016	0.022
7(C)	**0.064**	0.014	0.039
8(C)	0.013	0.055	0.034
9(C)	0.029	0.023	0.026
10(C)	0.021	0.019	0.020
11(C)	0.024	0.031	0.028
12(H)	0.019	0.022	0.021

13(C)	0.018	0.035	0.027
14(H)	0.015	0.021	0.018
15(C)	0.017	**0.063**	0.040
16(C)	0.048	0.029	0.038
17(H)	0.025	0.023	0.024
18(C)	0.031	0.016	0.024
19(H)	0.019	0.045	0.032
20(C)	0.009	0.017	0.013
21(C)	0.030	0.000	0.015
22(C)	0.040	0.055	**0.048**
23(H)	0.024	0.031	0.028
24(C)	0.018	0.009	0.014
25(H)	0.013	0.007	0.010
26(C)	0.037	0.013	0.025
27(C)	0.011	0.009	0.010
28(H)	0.017	0.016	0.016
29(H)	0.012	0.013	0.013
30(C)	0.018	0.009	0.014
31(H)	0.013	0.007	0.010
32(C)	0.031	0.017	0.024
33(H)	0.019	0.014	0.017
34(C)	0.005	0.002	0.003
35(H)	0.009	0.004	0.007
36(O)	0.007	0.000	0.004
37(H)	0.011	0.012	0.012
38(H)	0.021	**0.101**	**0.061**
39(H)	0.012	0.010	0.011
40(H)	0.012	0.015	0.013
41(H)	0.011	0.021	0.016
42(H)	0.012	0.023	0.017

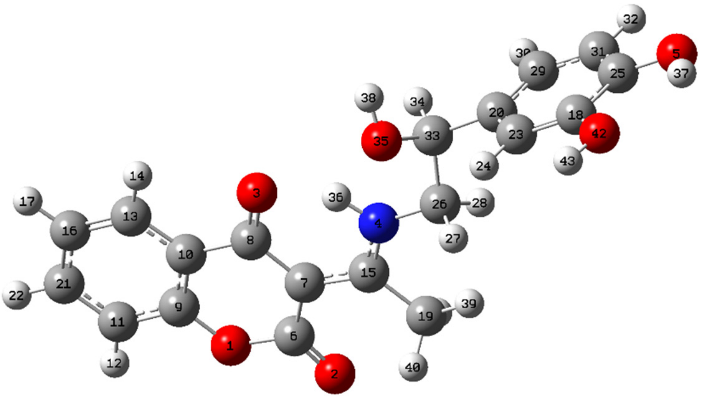

Figure S18. The atom numbering scheme for **L3**.

Table S14. CFFs for the specific atoms of **L3**.

Atom	f-	f+	f0
1(O)	0.024	0.017	0.020
2(O)	**0.077**	0.035	**0.056**
3(O)	0.025	**0.057**	0.041
4(N)	0.033	0.037	0.035
5(O)	**0.056**	0.016	0.036
6(C)	0.024	0.015	0.020
7(C)	**0.055**	0.013	0.034
8(C)	0.012	0.053	0.033
9(C)	0.026	0.022	0.024
10(C)	0.019	0.018	0.018
11(C)	0.022	0.031	0.026
12(H)	0.017	0.022	0.020
13(C)	0.016	0.035	0.025
14(H)	0.013	0.021	0.017
15(C)	0.015	**0.061**	0.038
16(C)	0.044	0.028	0.036
17(H)	0.023	0.022	0.022
18(C)	0.038	0.010	0.024
19(C)	0.008	0.017	0.012
20(C)	0.032	-0.002	0.015
21(C)	0.036	0.054	**0.045**
22(H)	0.022	0.031	0.026
23(C)	0.015	0.006	0.011
24(H)	0.014	0.023	0.018
25(C)	0.042	0.013	0.028
26(C)	0.010	0.009	0.009
27(H)	0.015	0.022	0.018
28(H)	0.011	0.012	0.011
29(C)	0.034	0.008	0.021
30(H)	0.019	0.006	0.012
31(C)	0.027	0.015	0.021
32(H)	0.021	0.012	0.017
33(C)	0.005	0.002	0.004
34(H)	0.010	0.005	0.007
35(O)	0.009	-0.001	0.004
36(H)	0.009	0.012	0.011
37(H)	0.022	0.025	0.023
38(H)	0.011	0.011	0.011
39(H)	0.010	0.015	0.012
40(H)	0.011	0.019	0.015
41(H)	0.011	0.023	0.017
42(O)	0.039	0.022	0.031
43(H)	0.020	**0.119**	**0.069**

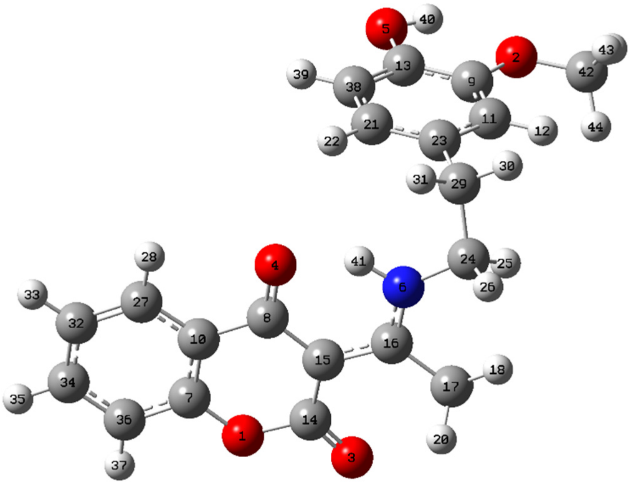

Figure S19. The atom numbering scheme for **L4**.

Table S15. CFFs for the specific atoms of **L4**.

Atom	f-	f+	f0
1(O)	0.020	0.019	0.019
2(O)	0.041	0.008	0.024
3(O)	**0.063**	0.040	**0.052**
4(O)	0.014	**0.066**	0.040
5(O)	**0.066**	0.013	0.039
6(N)	0.020	0.046	0.033
7(C)	0.019	0.026	0.022
8(C)	0.009	**0.063**	0.036
9(C)	0.045	0.009	0.027
10(C)	0.011	0.021	0.016
11(C)	0.021	0.005	0.013
12(H)	0.019	0.009	0.014
13(C)	**0.051**	0.011	0.031
14(C)	0.020	0.017	0.019
15(C)	0.044	0.015	0.029
16(C)	0.011	**0.072**	**0.042**
17(C)	0.007	0.020	0.013
18(H)	0.010	0.019	0.014
19(H)	0.008	0.028	0.018
20(H)	0.012	0.022	0.017
21(C)	0.043	-0.006	0.019

22(H)	0.020	-0.003	0.009
23(C)	0.049	-0.005	0.022
24(C)	0.011	0.013	0.012
25(H)	0.009	0.014	0.012
26(H)	0.018	0.036	0.027
27(C)	0.011	0.040	0.025
28(H)	0.009	0.024	0.016
29(C)	0.010	0.007	0.009
30(H)	0.019	0.031	0.025
31(H)	0.014	0.008	0.011
32(C)	0.034	0.032	0.033
33(H)	0.019	0.025	0.022
34(C)	0.030	0.062	**0.046**
35(H)	0.019	0.035	0.027
36(C)	0.019	0.035	0.027
37(H)	0.015	0.025	0.020
38(C)	0.029	0.004	0.016
39(H)	0.023	0.005	0.014
40(H)	0.026	0.009	0.017
41(H)	0.004	0.014	0.009
42(C)	0.015	0.011	0.013
43(H)	0.016	0.022	0.019
44(H)	0.014	0.008	0.011
45(H)	0.019	0.022	0.021

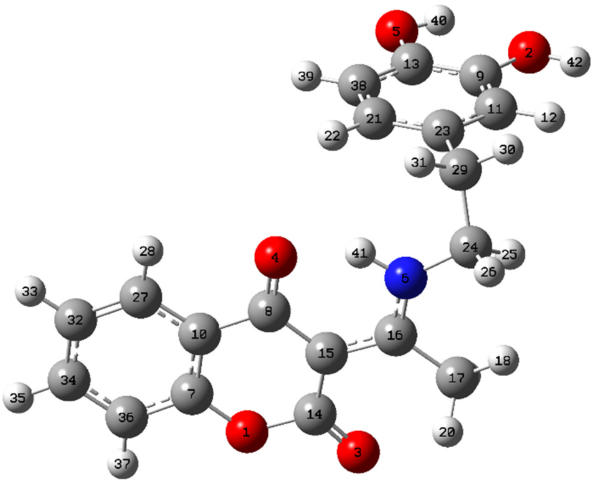

Figure S20. The atom numbering scheme for **L5**.

Table S16. CFFs for the specific atoms of **L5**.

Atom	f-	f+	f0
1(O)	0.022	0.018	0.020
2(O)	0.047	0.018	0.032
3(O)	**0.069**	0.037	**0.053**
4(O)	0.017	**0.060**	0.038
5(O)	**0.064**	0.014	0.039
6(N)	0.024	0.041	0.033
7(C)	0.022	0.024	0.023
8(C)	0.009	0.058	0.034
9(C)	0.048	0.010	0.029
10(C)	0.013	0.019	0.016
11(C)	0.022	0.008	0.015
12(H)	0.021	0.029	0.025
13(C)	**0.049**	0.011	0.030
14(C)	0.022	0.016	0.019
15(C)	0.047	0.014	0.030
16(C)	0.012	**0.066**	0.039
17(C)	0.007	0.018	0.013
18(H)	0.011	0.017	0.014
19(H)	0.009	0.026	0.017
20(H)	0.012	0.021	0.016
21(C)	0.039	-0.004	0.018
22(H)	0.018	-0.002	0.008
23(C)	0.045	-0.004	0.021
24(C)	0.011	0.011	0.011
25(H)	0.010	0.012	0.011
26(H)	0.019	0.031	0.025
27(C)	0.013	0.037	0.025
28(H)	0.010	0.022	0.016
29(C)	0.010	0.007	0.008
30(H)	0.018	0.026	0.022
31(H)	0.014	0.008	0.011
32(C)	0.038	0.030	0.034
33(H)	0.020	0.024	0.022
34(C)	0.033	0.058	**0.045**
35(H)	0.020	0.033	0.027
36(C)	0.021	0.033	0.027
37(H)	0.016	0.023	0.020
38(C)	0.026	0.006	0.016
39(H)	0.021	0.007	0.014
40(H)	0.026	0.014	0.020
41(H)	0.005	0.012	0.009
42(H)	0.025	**0.080**	**0.052**

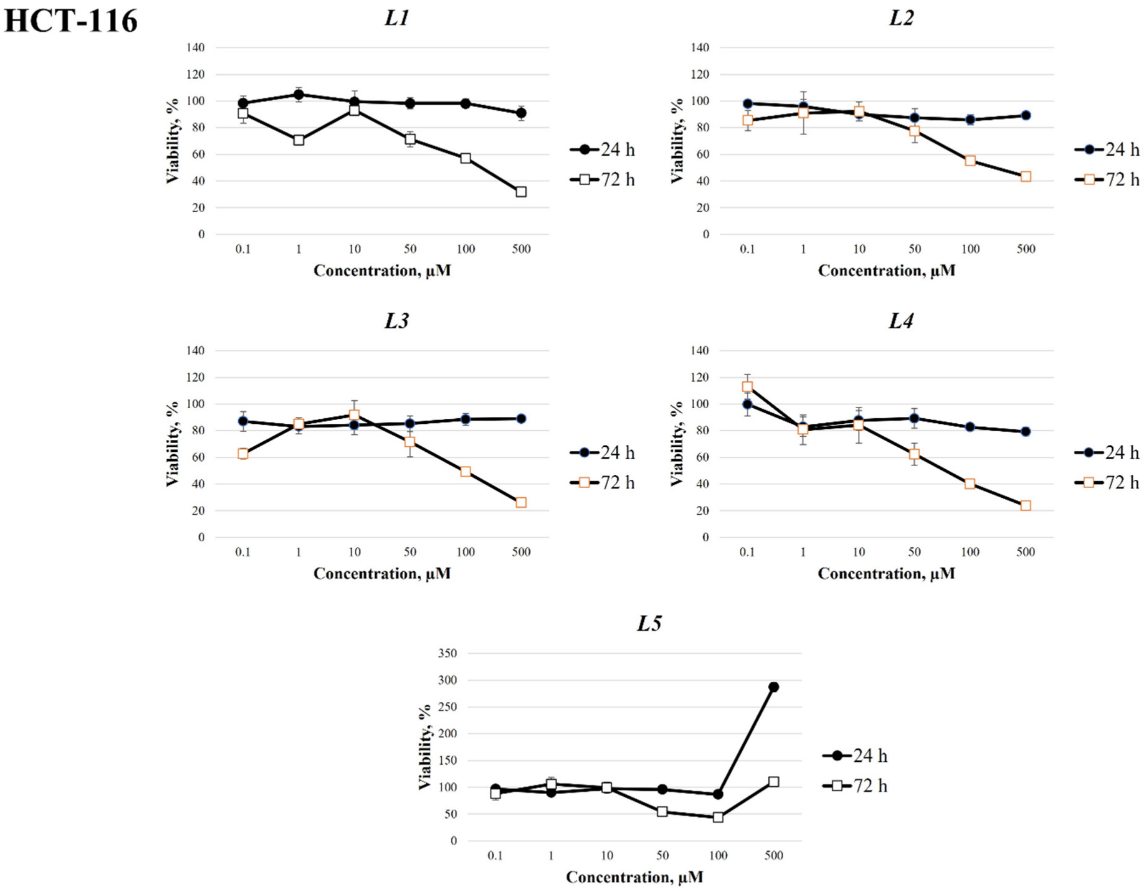

Figure S21. The dose response curves of the effect of **L1**, **L2**, **L3**, **L4**, and **L5** on HCT-116 cell line growth after 24 and 72 h of exposure.

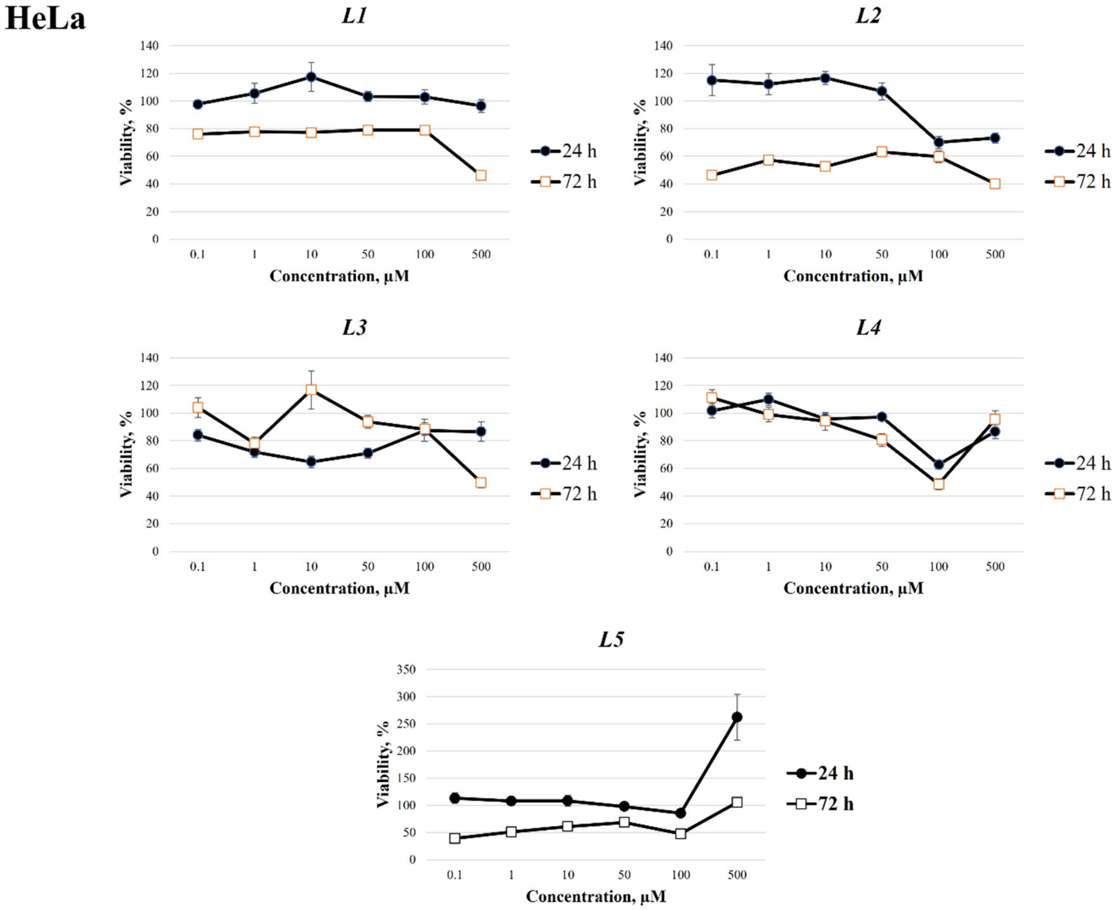

Figure S22. The dose response curves of the effect of **L1**, **L2**, **L3**, **L4**, and **L5** on HeLa cell line growth after 24 and 72 h of exposure.

MDA-MB-231

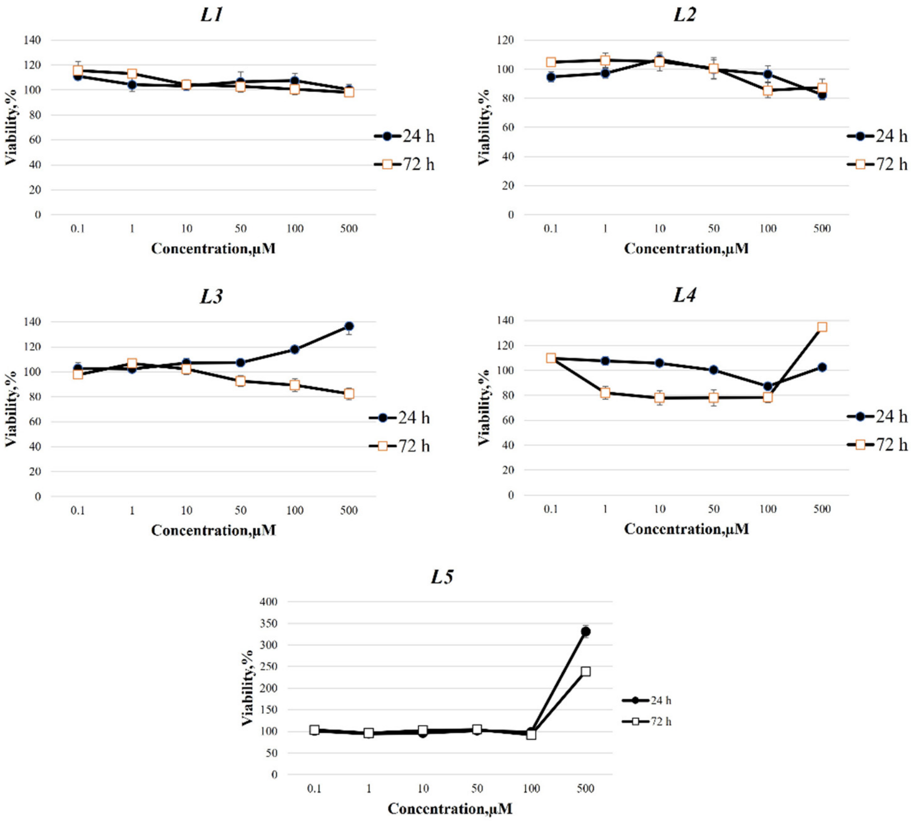

Figure S23. The dose response curves of the effect of **L1**, **L2**, **L3**, **L4**, and **L5** on MDA-MB-231 cell line growth after 24 and 72 h of exposure.

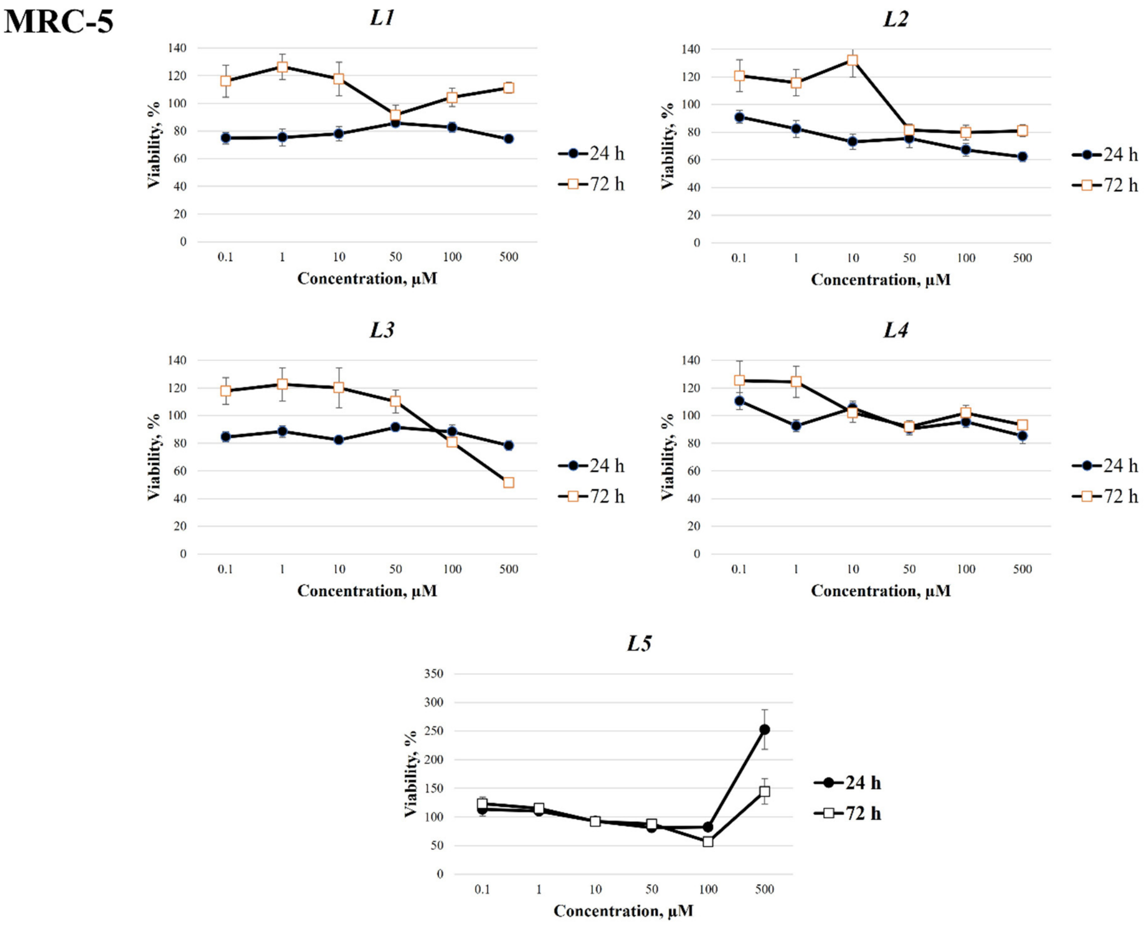

Figure S24. The dose response curves of the effect of **L1**, **L2**, **L3**, **L4**, and **L5** on MRC-5 cell line growth after 24 and 72 h of exposure.

Table S17. Selectivity index of tested substances after 24 and 72 h exposure.

**SI**	HCT-116/MRC-5		HeLa/MRC-5	
	24 h	72 h	24 h	72 h
**L1**	1	2.9	1	1.1
**L2**	1	2.0	1	2.4
**L3**	1	4.7	1	1
**L4**	1	6.8	1	3.3
**L5**	1	6.8	1	5.4

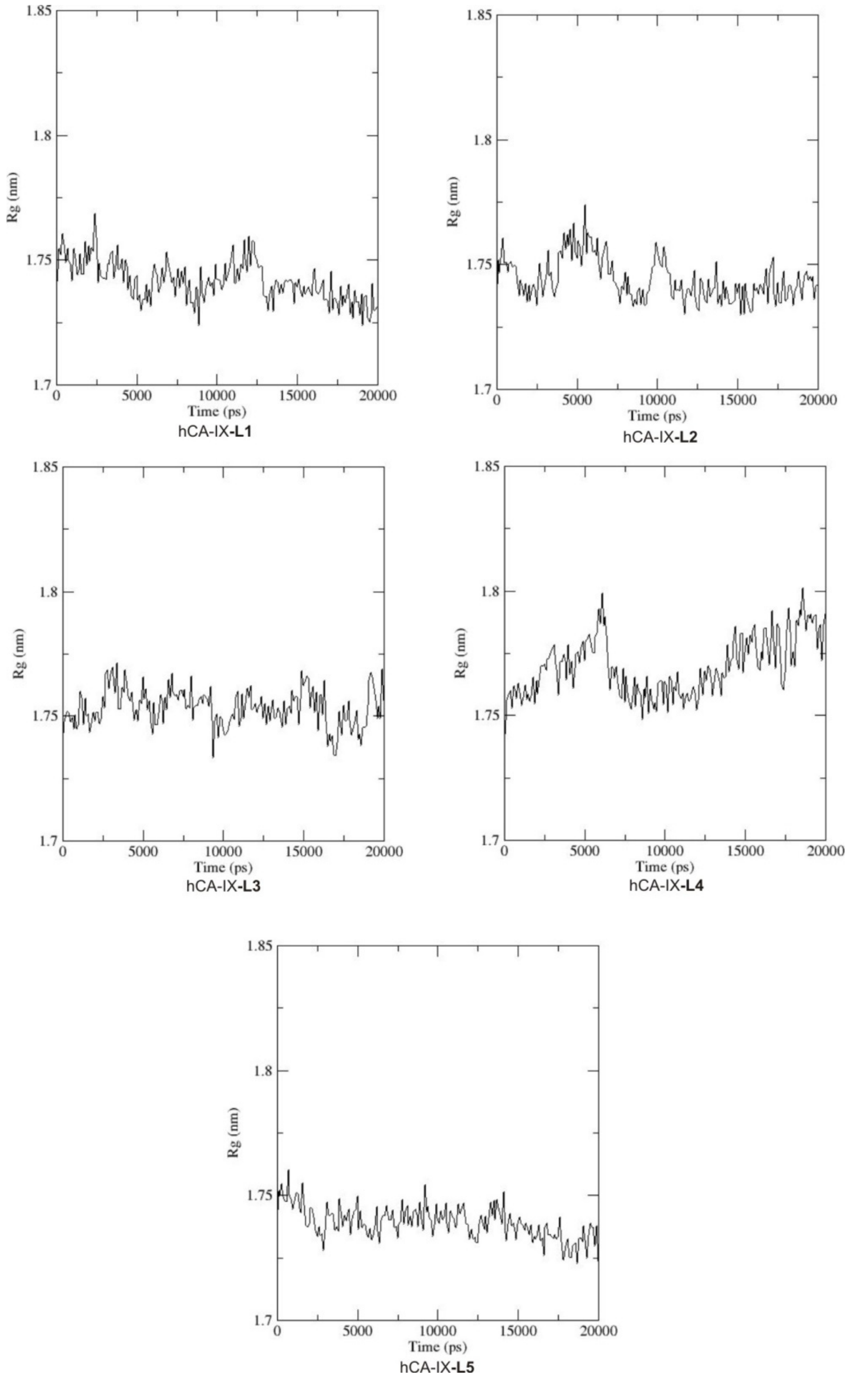

Figure S25. The plot of the radius of gyration (Rg) during 20 ns MD simulation of hCA-IX in complex with all candidate compounds.

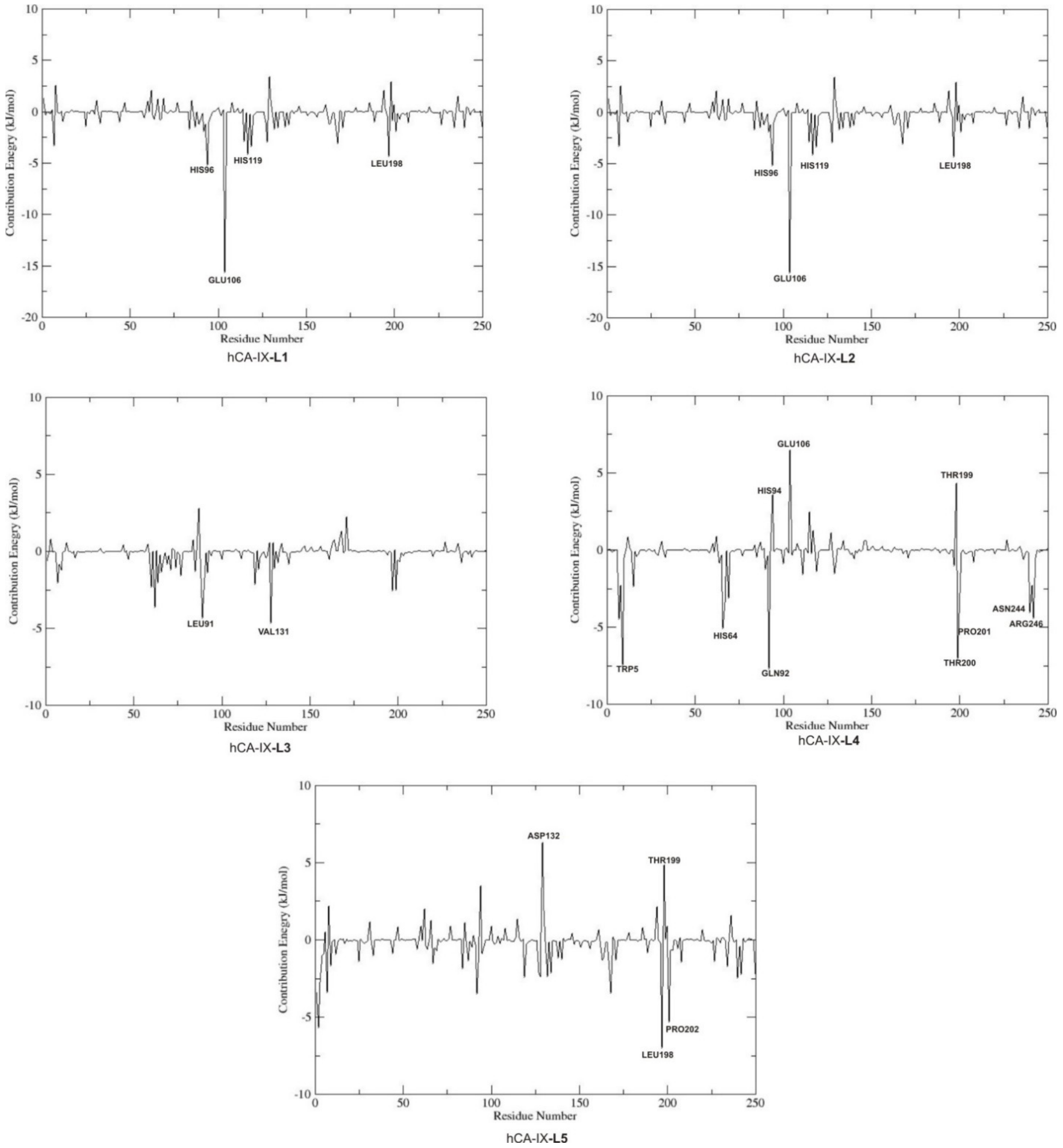

Figure S26. Comparison of the contribution of hCA-IX residues to the binding free energy in complexes with investigated ligands.

Table S18. The Lipinski rules applied to coumarin-neurotransmitter derivatives.

Compound	Molecular weight (<500 DA)	Log P (logP ≤ 5)	H bond donors (≤ 5)	H bond acceptors (≤ 10)	Number of violations
**L1**	323.3	1.90	2	4	0
**L2**	339.3	1.80	3	5	0
**L3**	355.3	0.86	4	6	0
**L4**	353.4	1.84	2	5	0
**L5**	339.3	1.56	3	5	0
